# Dinosaur paleohistology: review, trends and new avenues of investigation

**DOI:** 10.7717/peerj.7764

**Published:** 2019-09-27

**Authors:** Alida M. Bailleul, Jingmai O’Connor, Mary H. Schweitzer

**Affiliations:** 1 Key Laboratory of Vertebrate Evolution and Human Origins of the Chinese Academy of Sciences, Institute of Vertebrate Paleontology and Paleoanthropology, Beijing, China; 2 CAS Center for Excellence in Life and Paleoenvironment, Beijing, China; 3 Department of Biology, North Carolina State University, Raleigh, NC, USA; 4 North Carolina Museum of Natural Science, Raleigh, NC, USA; 5 Department of Geology, Lund University, Lund, Sweden; 6 Museum of the Rockies, Montana State University, Bozeman, MT, USA

**Keywords:** Dinosaurs, Birds, Mineralized tissues, Soft-tissues, Molecular paleontology, Paleohistochemistry, Standard paleohistology, New trends

## Abstract

In the mid-19th century, the discovery that bone microstructure in fossils could be preserved with fidelity provided a new avenue for understanding the evolution, function, and physiology of long extinct organisms. This resulted in the establishment of paleohistology as a subdiscipline of vertebrate paleontology, which has contributed greatly to our current understanding of dinosaurs as living organisms. Dinosaurs are part of a larger group of reptiles, the Archosauria, of which there are only two surviving lineages, crocodilians and birds. The goal of this review is to document progress in the field of archosaur paleohistology, focusing in particular on the Dinosauria. We briefly review the “growth age” of dinosaur histology, which has encompassed new and varied directions since its emergence in the 1950s, resulting in a shift in the scientific perception of non-avian dinosaurs from “sluggish” reptiles to fast-growing animals with relatively high metabolic rates. However, fundamental changes in growth occurred within the sister clade Aves, and we discuss this major evolutionary transition as elucidated by histology. We then review recent innovations in the field, demonstrating how paleohistology has changed and expanded to address a diversity of non-growth related questions. For example, dinosaur skull histology has elucidated the formation of curious cranial tissues (e.g., “metaplastic” tissues), and helped to clarify the evolution and function of oral adaptations, such as the dental batteries of duck-billed dinosaurs. Lastly, we discuss the development of novel techniques with which to investigate not only the skeletal tissues of dinosaurs, but also less-studied soft-tissues, through molecular paleontology and paleohistochemistry—recently developed branches of paleohistology—and the future potential of these methods to further explore fossilized tissues. We suggest that the combination of histological and molecular methods holds great potential for examining the preserved tissues of dinosaurs, basal birds, and their extant relatives. This review demonstrates the importance of traditional bone paleohistology, but also highlights the need for innovation and new analytical directions to improve and broaden the utility of paleohistology, in the pursuit of more diverse, highly specific, and sensitive methods with which to further investigate important paleontological questions.

## Introduction

Histology, from the Greek *histos*, meaning “web” or “woven material,” is the study of tissues at the microscopic, or sub-microscopic level. Histology is widely utilized by many sub-disciplines of biology, such as pathology and veterinary medicine (Musumeci, 2014), as well as paleontology (Padian & Lamm, 2013), to reveal cellular and extracellular structures that cannot be deciphered or understood at the gross, morphological scale, but which reveal important aspects of an organism’s biology.

**Figure 1:**
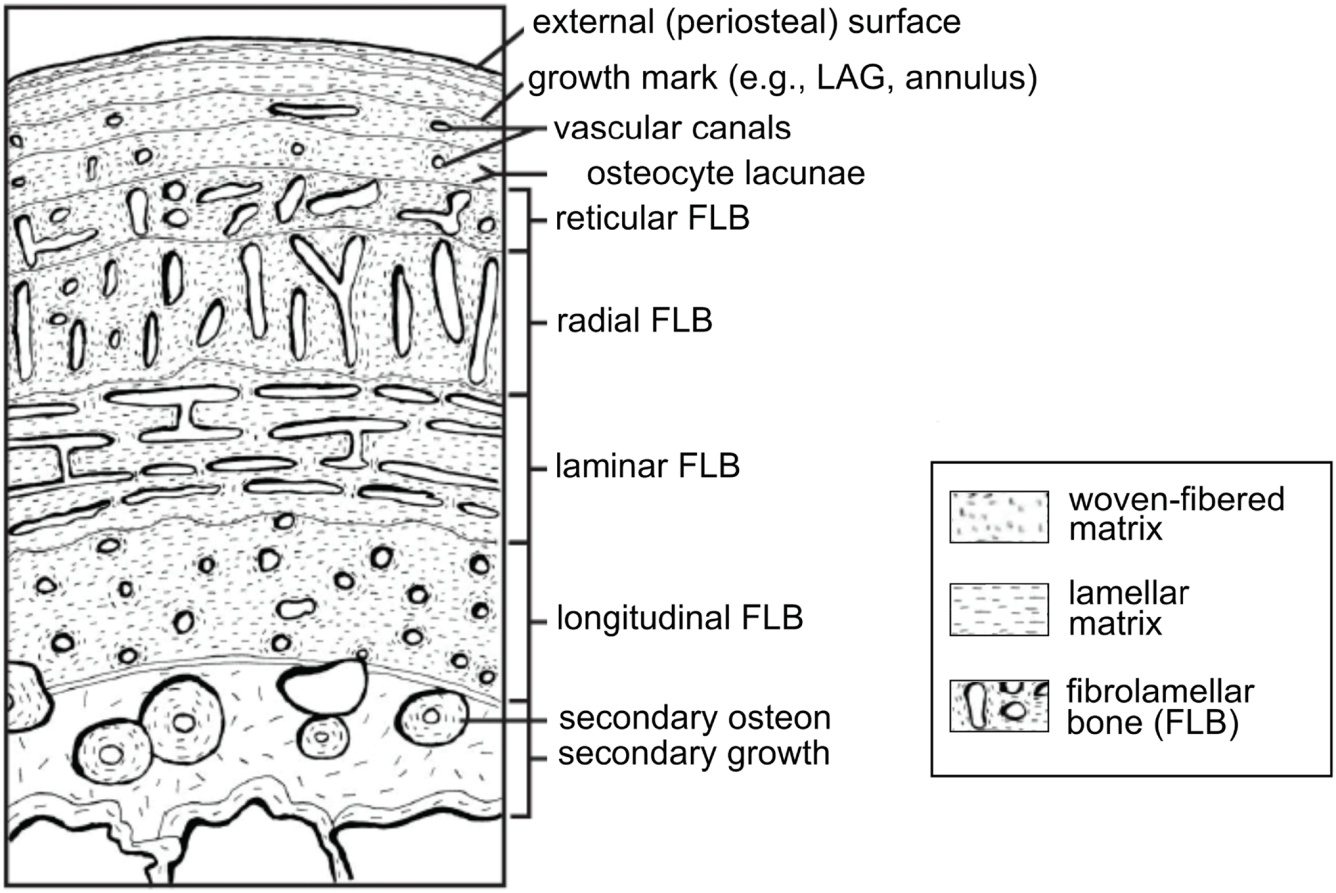
Schematic representation of the cortex of a long bone seen in cross-section. The degree of vascularization, the orientation of vascular canals (i.e., longitudinal, laminar, radial, or reticular orientations), and the degree of organization of the collagenous matrix, e.g., woven, lamellar (or parallel-fibered, not shown here) are a direct reflection of the local bone growth rate. When growth slows down and/or stops completely, which typically occurs annually, it is marked by a line of arrested growth (LAG) or other types of growth marks. Note that the schematized structures may not all necessarily be found together within a single section. From Huttenlocker, Woodward & Hall (2013); reproduced with permission; © 2013 by the Regents of the University of California. Published by the University of California Press.

Vertebrate organisms are hierarchical: molecules (DNA, proteins, fatty acids, carbohydrates) combine to form functioning cells, which in turn combine to form distinct tissues with differing functions. These tissues are organized to form the organs and organ systems of any functioning organism. By definition, a tissue is a material comprised of one particular cell type that has a single function (Mescher, 2013), and based upon function, micromorphology, and chemical composition, are classified into four basic types: nervous, muscle, epithelial, and connective tissues. Paleontologists are most familiar with skeletal tissues, which are types of connective tissues, and include bone (Fig. 1), calcified cartilage, and dental tissues. Skeletal tissues are mineralized and thus favored to enter the rock record over those unmineralized “soft” tissues such as muscles, nerves, and the majority of connective tissues (e.g., tissues within the integument).

## Survey methodology

Paleohistology focuses on the study of the microstructure of fossilized skeletal tissues (Francillon-Vieillot et al., 1990), but recently has been applied to traces of originally unbiomineralized soft-tissues (Schweitzer, Wittmeyer & Horner, 2005; Schweitzer et al., 2005a; Chin et al., 2003; McNamara et al., 2009; Lindgren et al., 2018). Histology has been used to study a great variety of extinct organisms, a complete review of which would be a monumental task; instead, we focus on the literature pertaining to a single group, the Archosauria, to illustrate the utility of paleohistology and recent growth in its applications and methodologies. The Archosauria is a vast clade that includes the Crurotarsi (all taxa more closely related to crocodiles than to birds) and the Ornithodira (the clade that contains pterosaurs and dinosaurs including birds). Within this diverse clade, here we concentrate on studies conducted on the Dinosauria, to which the greatest diversity of paleohistological techniques have been applied. The extant phylogenetic bracket of non-avian dinosaurs, formed of crown birds and crocodilians (Witmer, 1995; Bryant & Russell, 1995) informs on interpretations of bone microstructure and we discuss this concept as it applies to paleohistology.

## Inaugural studies on fossil bone and fossil archosaurs

The primary methodology employed in paleohistology is a modification of petrographic ground-section methods used by petrologists to study the microcrystalline structure of rocks (Lamm, 2013). Interestingly, the first petrographic ground sections ever made were not made from rock, but from a fossil tree (Falcon-Lang & Digrius, 2014). In 1828, two British scientists, Henry Witham and William Nicol, experimented by grinding sheets of a newly discovered petrified tree trunk, and their successful protocol was rapidly adopted by geologists and applied to the fields of petrology and mineralogy (Falcon-Lang & Digrius, 2014).

A few years later, this technique was applied to fossil vertebrates. Agassiz (1833–1843), Gross (1934), and Ørvig (1951) examined Paleozoic jawless vertebrates and early gnathostomes, but—because of its inherently destructive nature—it took more than a hundred years before this technique became common practice and available in paleohistological laboratories world-wide. To our knowledge, the first microscopic thin-sections of fossil archosaurs were made from material belonging to the ankylosaur *Hylaeosaurus* (Mantell, 1850a), the titanosaur *Pelorosaurus* (Mantell, 1850b), an indeterminate hadrosaur (i.e., “*Trachodon*,” a genus no longer considered valid; Seitz, 1907), and some juvenile hadrosaurs (Nopcsa, 1933). Quekett (1849) compared the histological structure of bone in four extant groups (mammals, birds, reptiles, and fish), and mentions briefly *Iguanodon* (stating that it does not look different from the bone of the “pigmy race of lizards that treads under our feet”). However, it is unclear whether he actually made a thin-section of *Iguanodon* (Quekett, 1849), and therefore, to our knowledge, the first clear description of dinosaur bone microstructure are those of British paleontologist Gideon Mantell (1850a, 1850b). Mantell provides a drawing of a thin section from the “dorsal dermal spine” of *Hylaerosaurus*, and refers to the structures as “decussating ossified fibers,” “bone-cells,” and “Haversian canals” (Mantell, 1850a). In his other work from the same year on *Pelorosaurus* (Mantell, 1850b), even though he does not provide a drawing made at high magnification, he describes a transverse thin section from a humerus, and notes that the bone exhibits an “intimate structure beautifully preserved; the bone cells, and Haversian canals, are as distinct as in recent bones.” Therefore, for almost 170 years it has been known that original histological and cellular structures can be preserved intact in fossil archosaurs.

Despite this knowledge, it was not until much later that the systematic study of archosaur paleohistology began in earnest with the pioneering studies of Enlow & Brown (1956, 1957, 1958). They described the microstructure of limb and skull bones from a number of extant and fossil birds, crocodilians, and some iconic dinosaurs including *Allosaurus*, *Brachiosaurus*, and *Iguanodon*. These early works further demonstrated that histological and cellular structure preserves intact in fossil archosaur bone, additionally revealing that fossil bone contained a wealth of information regarding the evolution and function of skeletal tissues in extinct organisms (these studies also confirmed observations made on non-archosaur fossil bone, from much more recent organisms—see Stout, 1978 for an excellent review).

In the 1960s and 1970s, Armand de Ricqlès built upon these seminal works, describing the bone microstructure of numerous fossil vertebrates (De Ricqlès, 1968, 1969, 1972, 1974, 1975, 1976, 1980, 1981). His original goal was to discover diagnostic phylogenetic characters that could be used to classify fragmentary fossil remains (De Ricqlès, 2011). He examined numerous fossil groups, including amphibians (e.g., members of Stegocephalia), some early amniotes, mesosaurs, and archosaurs (i.e., basal sauropodomorphs, sauropods), but rapidly realized that the microstructure of bony tissues may not be a good indicator of phylogeny (but see Garilli et al., 2009). Instead, he advanced the idea that paleohistological features could be correlated with growth rates and thus could indirectly shed light on the thermal physiology of extinct organisms (De Ricqlès, 1968, 1969, 1974, 1975, 1980, 1981).

He based this reasoning on the neontological observations of Rodolfo Amprino, who showed that local bone microstructure (Fig. 1) reflects growth rate (Amprino, 1947; also see De Margerie, Cubo & Castanet, 2002). De Ricqlès (1980) was the first to apply this to fossil bone, deducing that organisms showing higher degrees of vascularization grew at higher rates than those with fewer vascular canals. Similarly, he showed that the degree of organization of the collagen fibers in bone may also be used to approximate differences in local growth rates (Fig. 1). He hypothesized that woven bone, with the lowest degree of organization, would exhibit the fastest growth, whereas the highly organized lamellar bone would form more slowly (see Francillon-Vieillot et al., 1990). His early histological examinations of dinosaur bones suggested that they did not grow in a manner similar to extant cold-blooded, slow-growing reptiles which deposit poorly vascularized cortical bone, interrupted by many lines of arrested growth (LAGs) (such as extant crocodiles, who can take 20–30 years to reach skeletal maturity; Chabreck & Joanen, 1979). De Ricqlès (1968, 1972, 1980) concluded that dinosaurs had a physiology that more closely approximated that of extant, fast-growing, endothermic birds.

This qualitative (or semi-quantitative) relationship between bone vascularization, collagen fiber orientation, growth rate, and physiology is still used today and is widely accepted to be correlated with an animal’s metabolic rate. However, it was only very recently that these relationships were tested statistically (Montes et al., 2007). Currently, paleohistological examinations (whether qualitative, semi-quantitative, or quantitative) are the most accurate tool available for making inferences regarding growth rates in fossil vertebrates.

These fundamental works demonstrated the relevance of paleohistological analyses to broader evolutionary questions, and were integral in bringing histological analyses into the mainstream of vertebrate paleontology. The growth and physiology studies conducted by de Ricqlès et al. opened a new path for paleohistology and this work continues to influence the field today.

## The “growth age” of dinosaur histology

### Growth and physiology

#### Dinosaur growth and physiology

The pioneering studies of Enlow & Brown, (1957), De Ricqlès (1969), and Enlow (1969) led to a plethora of histological studies designed to infer growth and maturity in fossil archosaurs. Such studies, beginning in earnest in the 1970s (De Ricqlès, 1972, 1975, 1980; Chinsamy, 1993; Varricchio, 1993; Reid, 1996; Horner, De Ricqlès & Padian, 1999, 2000; Sander, 2000a; Sander et al., 2004, 2006; Chinsamy & Elzanowski, 2001; De Ricqlès, Padian & Horner, 2001; Padian, De Ricqlès & Horner, 2001; Horner & Padian, 2004; Bybee, Lee & Lamm, 2006; Klein & Sander, 2007), continue to the present (Stein et al., 2010; Sander et al., 2011; Woodward, Horner & Farlow, 2011; Farke et al., 2013; Griebeler, Klein & Sander, 2013; Dumont et al., 2014; Vanderven, Burns & Currie, 2014; Rogers et al., 2016; Mitchell, Sander & Stein, 2017; Woodward, 2019).

It was not until the 1990s that major attempts to reconstruct dinosaurian growth rates and growth curves began. Chinsamy (1993) was the first to attempt reconstruction of a dinosaur growth curve with her study of the sauropodomorph *Massospondylus*. Around the same time, collaborations between de Ricqlès, Horner, and Padian produced significant advances in our current understanding of archosaur growth (Horner & Currie, 1994; Horner, De Ricqlès & Padian, 1999, 2000; Horner & Padian, 2004; Padian, De Ricqlès & Horner, 2001, Padian, Horner & De Ricqlès, 2004; De Ricqlès et al., 2001, 2008) through pivotal studies, in which they sampled the limb bones (both diaphyseal cross-sections and epiphyseal longitudinal sections) of embryos and hatchlings of extant crocodiles, turtles, and birds and compared them to similarly prepared material from dinosaur embryos, including *Troodon*, *Maiasaura*, and *Hypacrosaurus* (Horner, Padian & De Ricqlès, 2001; Horner & Currie, 1994; also see the more recent study by Reisz et al., 2013 on sauropodomorph embryos). These very young dinosaurs showed strong similarities in both diaphyseal vascularization and epiphyseal micromorphologies (i.e., a thickened zone of hypertrophic chondrocytes; also found by Barreto et al., 1993) intermediate between non-avian dinosaurs and birds during early ontogenetic stages, reflecting in part their close phylogenetic affinities. This was followed by examination of many other species and specimens, including ontogenetically older specimens of *Hypacrosaurus* and *Maiasaura* (Horner, De Ricqlès & Padian, 1999, 2000), the iconic *Tyrannosaurus rex* (Erickson et al., 2004; Horner & Padian, 2004) and hypsilophodonts (Horner et al., 2009).

Other investigators contributed to the growing literature on paleohistology, broadening the scope of these investigations. Most notably, considerable work has been done to understand the histology and growth strategies of sauropodomorphs, including sauropod dinosaurs, the largest terrestrial animals that ever lived (Sander, 2000a; Sander et al., 2004, 2006, 2011; Rogers & Erickson, 2005; Klein & Sander, 2007, 2008; Klein et al., 2012; Stein et al., 2010; Dumont et al., 2011; Griebeler, Klein & Sander, 2013; Hofmann, Stein & Sander, 2014; Mitchell & Sander, 2014; Mitchell, Sander & Stein, 2017). Other works on archosaurs included histological studies of pterosaurs (the sister group to dinosaurs; e.g., De Ricqlès et al., 2000; Padian, Horner & De Ricqlès, 2004) basal archosaurs, and even more basal Archosauriformes including key species such as *Erythrosuchus* and *Euparkeria* (De Ricqlès et al., 2008).

Growth strategies in extinct dinosaurs are not simply understood by analyzing collagen fiber orientations or the degree of vascularization, but also by quantitatively analyzing preserved annual growth marks (i.e., LAGs, see below). Growth marks are used to establish individual growth curves and to statistically test the best-fitting growth model (based on models successfully applied to extant species; e.g., see Erickson & Tumanova, 2000; Erickson, Rogers & Yerby, 2001; Lehman & Woodward, 2008; Griebeler, Klein & Sander, 2013). Growth model fitting is a recent quantitative approach in paleohistology to the study of dinosaur life history (Erickson & Tumanova, 2000; Erickson, Rogers & Yerby, 2001; Lehman & Woodward, 2008; Griebeler, Klein & Sander, 2013). The larger the sample size is, the more accurate the model is. For example, Woodward et al. (2015) used 50 tibiae to reconstruct the growth curve of the hadrosaur *Maiasaura*, utilizing more data than any previous growth study. This study represents the most accurate growth dynamic reconstruction of a dinosaur—or in fact, of any fossil vertebrate—to date (but see Bybee, Lee & Lamm, 2006 for another relevant study with a large sample size). Woodward et al. (2015) determined that *Maiasaura* would have reached sexual maturity in 3 years and skeletal maturity after 8 years, contributing to growing data supporting the idea that dinosaurs grew much faster than living reptiles.

Beyond species level growth reconstructions, paleohistology can be utilized at a much larger scale, possibly representing the best available tools with which to elucidate major evolutionary transitions in growth rates and physiology (Padian, De Ricqlès & Horner, 2001; De Ricqlès et al., 2008). Since the pioneering studies of De Ricqlès (1980, 1981) using histology to propose the endothermy of dinosaurs, histology has clarified many aspects of the transition from ectothermy to endothermy, and new methods have been developed (Cubo et al., 2012; Legendre et al., 2016; Myhrvold, 2016). For example, Cubo et al. (2012) conducted the first quantitative study using a paleobiological model to estimate bone growth rates (i.e., local cortical bone apposition rates) in fossils. This study showed that during the evolution of archosaurs, bone growth rates increased from the last common ancestor of Ornithodira to extant birds, but decreased from the last common ancestor of Crurotarsi to extant crocodiles (Cubo et al., 2012). Based on the equations of Montes et al. (2007), Legendre et al. (2016) hypothesized that archosaurs have a high ancestral metabolic rate and that the ectothermic physiology seen in extant crocodiles is actually a derived condition (also see Grigg & Kirshner, 2015).

Note, however, that the dichotomy of ectothermic vs endothermic dinosaurs is overly simplistic (Grady et al., 2014; Werner & Griebeler, 2014; Legendre et al., 2016). Currently, the precise type and term for the physiology of extinct dinosaurs is still debated, as it has been recently proposed that they could have been mesotherms (Grady et al., 2014), or fast-growing ectotherms (Werner & Griebeler, 2014). Certainly a range of physiologies were present given the great diversity of this clade.

The evolution of growth strategies in dinosaurs during the “dinosaur-bird” transition has also been heavily studied (Padian, De Ricqlès & Horner, 2001; Erickson, Rogers & Yerby, 2001), which we will elaborate on in the following section.

#### The evolution of avian growth

Not all dinosaur clades exhibit the same growth strategy, and considerable differences exist in both extinct and extant groups. These differences are partially linked to the extreme variation in size and terrestrial lifestyles observed in different groups of non-avian dinosaurs. However, in early birds, changes are undoubtedly related to the evolution of flight. Histological data suggest that Mesozoic dinosaurs took several years to reach adult size (e.g., 8 years for Maiasaura; Woodward et al., 2015; up to 40 years for sauropods, Lehman & Woodward, 2008; Rogers & Erickson, 2005; Griebeler, Klein & Sander, 2013; Waskow & Sander, 2014). All data thus far indicates non-ornithuromorph dinosaurs reached sexual maturity prior to skeletal maturity (Sander, 2000a; Erickson et al., 2009; O’Connor et al., 2014; Woodward et al., 2015) in a pattern similar to extant crocodilians (Chabreck & Joanen, 1979; Woodward, Horner & Farlow, 2011).

This type of growth is opposite to the condition in extant birds, which nearly all reach skeletal maturity rapidly (within a year) and prior to sexual maturity, that we can refer to as “avian-style” growth (Ricklefs, 1968; Castanet et al., 2000; Ponton et al., 2004). The only exception are the flightless paleognaths, which can take between 3 and 9 years to reach full size (Lee & Werning, 2008; Bourdon et al., 2009). Histology indicates that the dinosaurs most closely related to Aves were more similar to birds in terms of growth strategies than previously acknowledged, and in fact Mesozoic theropod dinosaurs exhibited metabolic rates very close to those found in modern birds (Legendre et al., 2016).

**Figure 2:**
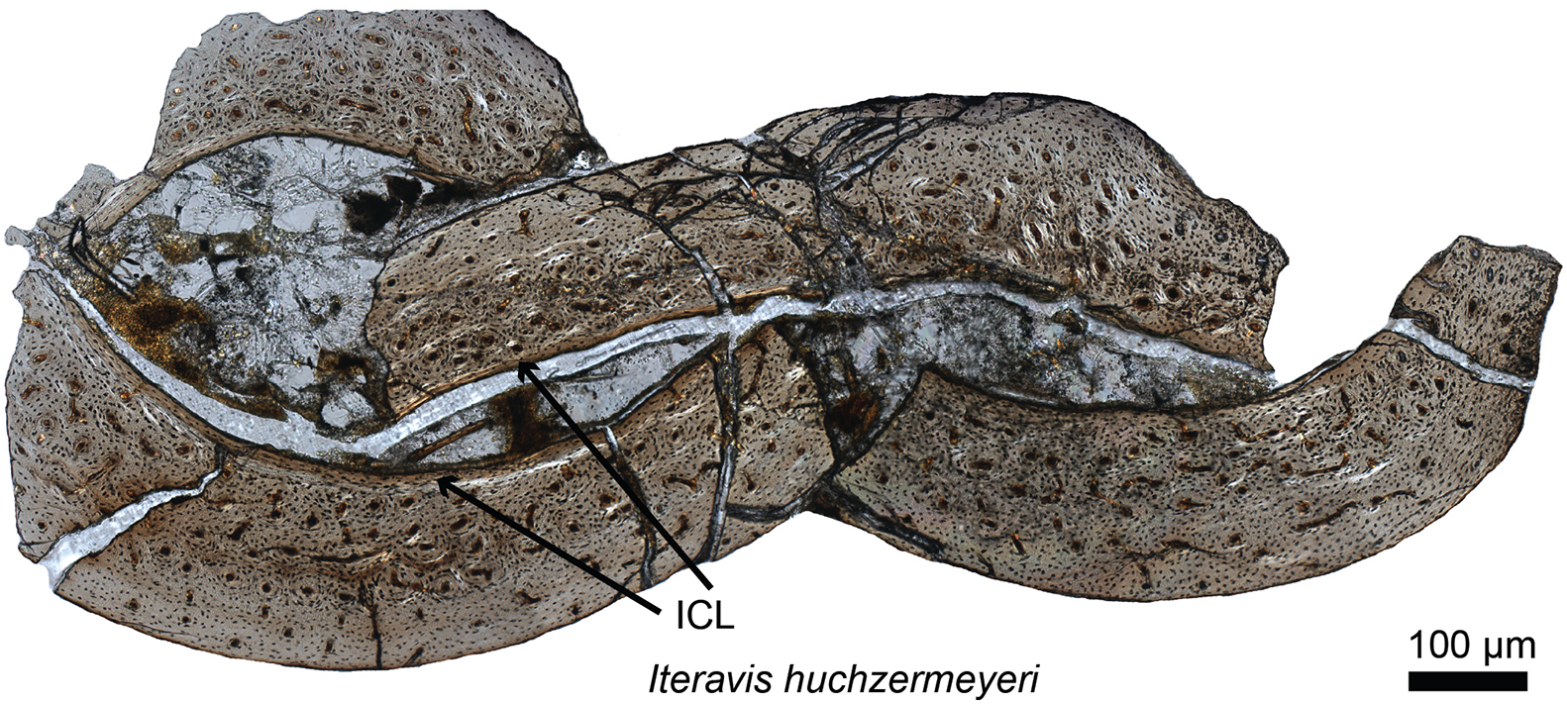
Petrographic ground section of the ulna of *Iteravis huchzermeyeri* IVPP V18958. Its histology shows a fairly vascularized tissue and an internal circumferential layer (ICL), the latter being found in extant birds with nearly complete skeletal growth. This microstructure and the absence of an annual growth mark suggests that this specimen was most likely less than a year-old and had not fully reached skeletal maturity. This represents the growth pattern typically found in most living birds, and is unlike that of more basal birds or non-avian dinosaurs who took multiple years to reach full skeletal maturity. Modified from O’Connor et al. (2015).

The timing of the origin of this “avian-style” growth is still under investigation (Fig. 2), but it evidently was absent in most stem birds. Paleohistological studies on the Jurassic *Archaeopteryx* (Erickson et al., 2009) and non-ornithuromorph birds from the Early Cretaceous indicate that, like non-avian dinosaurs, they also required several years to reach adult size (Chinsamy, Chiappe & Dodson, 1995; Erickson et al., 2007; O’Connor et al., 2014). Numerous paravians have been sectioned, sampling most of the phylogeny across the dinosaur-bird transition, but until recently, avian-style growth has only been reported in the derived ornithurines *Hesperornis* and *Ichthyornis* (Chinsamy, Martin & Dobson, 1998). Examination of the non-ornithurine ornithuromorph *Iteravis* (Fig. 2; O’Connor et al., 2015) suggests that the growth strategy observed in extant birds evolved independently in the Early Cretaceous outside the Ornithurae (O’Connor et al., 2015) but still in the crownward lineage that includes all extant birds. Similar results were also found in the non-ornithurine ornithuromorph *Yanornis* (Wang et al., 2019). Several studies also indicate that most stem birds, like their non-avian dinosaurian relatives, achieved reproductive maturity prior to skeletal maturity (O’Connor et al., 2014, 2018).

Much of what is known about the transition from dinosaur to avian-style growth comes from specimens found either in the middle to Upper Jurassic Yanliao Biota or the Lower Cretaceous Jehol Biota, both known from northeastern China (Zhou, Barrett & Hilton, 2003). The oviraptorosaur *Caudipteryx* (Erickson et al., 2009), paravians including *Anchiornis* (Zheng et al., 2014; Prondvai et al., 2018), basal birds including *Sapeornis* (Gao et al., 2012), *Jeholornis* (Erickson et al., 2009; O’Connor et al., 2014; Prondvai et al., 2018), *Confuciusornis* (Zhang, Hou & Ouyang, 1998; De Ricqlès et al., 2003; Chinsamy et al., 2013), the ornithuromophs *Archaeorhynchus* (Wang & Zhou, 2017) and *Iteravis* (O’Connor et al., 2015), and several enantiornithines (O’Connor et al., 2014, 2018) together with Late Cretaceous ornithothoracines from Argentina (Chinsamy, Chiappe & Dodson, 1995) have contributed greatly to our overall understanding of the evolution of avian growth strategies. Despite this work, the study of stem avian histology is still at a relatively early stage compared to that of non-avian dinosaurs. Increased sampling across groups, within groups and species (e.g., from multiple specimens of *Archaeopteryx*, and Jehol taxa from which at least partial growth series can be obtained), and between various skeletal elements will continue to clarify our understanding of dinosaurian and avian growth (O’Connor et al., 2014).

### Recent advances in the maturity assessment of dinosaur specimens

#### Lines of arrested growth and skeletal maturity

Histological examinations allow the assessment of individual age, and skeletal and sexual maturity in fossil specimens. Age is assessed by counting annually deposited LAGs (Fig. 1) (Horner, De Ricqlès & Padian, 1999; Erickson & Tumanova, 2000; Woodward et al., 2015). In living animals, these reflect a seasonal slow-down or cessation of growth that occurs during the harshest season of the year (see the detailed description of skeletochronology and retrocalculation methods in Woodward, Padian & Lee, 2013). Early on LAGs were correlated to ectothermy such that the presence of LAGs in a specimen was once inferred to indicate an ectothermic metabolism, whereas the absence of LAGs would suggest endothermy (e.g., see Chinsamy, 2005). However, it has recently been shown that even some extant endotherms (i.e., some mammals) arrest their growth cyclically, resulting in the formation of LAGs, as part of a plesiomorphic thermometabolic strategy to conserve energy (Köhler et al., 2012; also see Sander & Andrassy, 2006 for another study on mammals). These data confirm the annual cyclicity of LAGs in extant endothermic mammals and indicate that their presence cannot be used as an argument for ectothermy in fossils (Köhler et al., 2012).

Currently, LAGs are the most accurate indicators of ontogenetic age in extinct vertebrate organisms, the best records to infer growth models (Erickson & Tumanova, 2000; Erickson, Rogers & Yerby, 2001; Lehman & Woodward, 2008; Griebeler, Klein & Sander, 2013) and the best indicators of skeletal maturity. Indeed, as an individual approaches maturity, the space between the LAGs in limb bones gradually diminishes as they approach the periosteal surface (as seen in Fig. 1) eventually becoming organized into an external fundamental system (EFS; Cormack, 1987) or outer circumferential layer (OCL; Ponton et al., 2004). The presence of an EFS/OCL reflects the slowing or cessation of growth, which in turn indicates the attainment of skeletal maturity. EFS have been reported in birds (Ponton et al., 2004) and more recently, in American alligators (Woodward, Horner & Farlow, 2011). EFSs have been described in a few dinosaur specimens (e.g., in some sauropodomorphs, ankylosaurs, stegosaurs, and hadrosaurs; Sander & Klein, 2005; Redelstorff et al., 2013; Stein, Hayashi & Sander, 2013; Woodward et al., 2015). This absence has often been interpreted as a marker of “indeterminate growth” (growth continuous throughout the animal’s life, e.g., Chinsamy, 1993). However, in a recent examination of a large sample of American alligators (animals often considered to possess “indeterminate growth”), Woodward, Horner & Farlow (2011) found EFSs in the oldest specimens. Indeterminate growth is no longer considered a valid term, and presumably in all reptiles (including dinosaurs) growth is determinate (e.g., see discussions in Sander, 2000a; Redelstorff & Sander, 2009; Sander et al., 2011; Redelstorff et al., 2013; Stein, Hayashi & Sander, 2013; Padian & Lamm, 2013). Woodward, Horner & Farlow (2011) and Woodward et al. (2015) demonstrated that the absence of an EFS in specimens of non-avian dinosaurs simply indicates that they died before reaching skeletal maturity.

Woodward (2019) also recently described a new type of cortical growth mark in *Maiasaura* that only occurs prior to the deposition of the first LAG under normal conditions. Referred to as localized vascular changes, these are unlike LAGs in that they are non-annual and are hypothesized to represent times of temporary but repeated stress (Woodward, 2019). They cannot be used to directly assess maturity and age assessment, as can LAGs, but they do hold potential to interpret other aspects of extinct vertebrate biology if they are further investigated in extant archosaurs and other dinosaur species.

#### Medullary bone and sexual maturity

Arguably, the most accurate way to assess sexual maturity in fossil dinosaurs and birds is to find skeletons in direct association with a clutch, or with unlaid eggs preserved internally (Sato et al., 2005; Erickson et al., 2007; Bailleul et al., 2019). However, medullary bone (MB), a female specific skeletal tissue formed during egg production, can also be used as a marker of reproductive activity (and thus reproductive maturity) if identified correctly. Today MB is only found in extant, reproductively active female birds (Schweitzer et al., 2007). This ephemeral skeletal tissue acts as a calcium reservoir, helping birds meet the high calcium demands imposed by eggshell formation (Bloom, Bloom & McLean, 1941; Dacke et al., 1993). Derived from the endosteum, MB can line the medullary cavities and cancellous spaces of the entire avian skeleton in some taxa (Canoville, Schweitzer & Zanno, 2019). In living birds, MB is estrogen dependent, forms extremely fast (although this might not have always been the case in stem taxa; see O’Connor et al., 2018; Bailleul et al., 2019), is high in calcium relative to other types of bone tissue, and is highly vascular, all which facilitate its function as an easily mobilized calcium reservoir (Bloom, Bloom & McLean, 1941; Taylor & Moore, 1953; Yamamoto et al., 2001).

Two recent studies have demonstrated the widespread presence of MB in Neornithes (Werning, 2018; Canoville, Schweitzer & Zanno, 2019). Canoville, Schweitzer & Zanno (2019) report that the skeletal distribution of MB is directly related to the distribution of red bone marrow, and inversely correlated to the combined skeletal distributions of pneumaticity and yellow bone marrow. They also confirmed that avian MB can be deposited within virtually all skeletal elements, including those within the skull.

**Figure 3:**
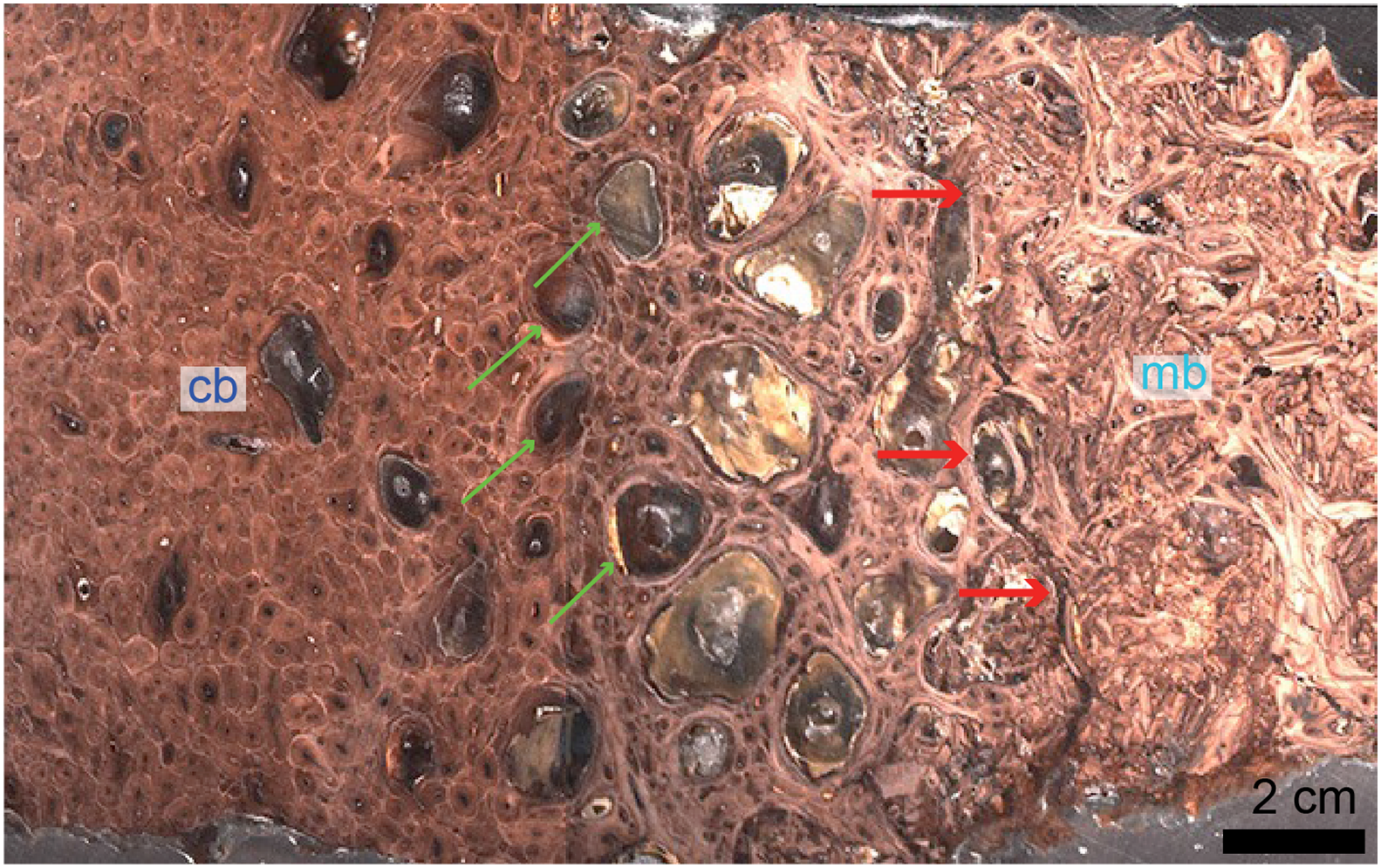
Petrographic ground section of a femur fragment of *Tyrannosaurus rex* (MOR 1125), showing cortical bone (cb) and medullary bone (mb). The cb shows many secondary osteons interspersed with increasingly large erosion rooms (green arrows). Red arrows show distinct boundary between cb and mb. Avian ****mb is found in reproductive female birds and is used as a calcium reservoir during eggshell formation. Since a similar tissue was found in this non-avian theropod dinosaur, it illustrated similarities between birds and dinosaurs at the microscopic scale and suggested that MOR 1125 was a gravid, female *T. rex*. This tissue has since been used to infer sexual maturity in other fossil archosaurs. Modified from Schweitzer et al. (2016).

Although the biology and chemistry of this tissue are now relatively well understood in living birds, the timing of the evolution of this tissue is still subject to debate. MB was first reported outside Aves in the femur (Fig. 3) and tibiae of a specimen of *T. rex* from Montana (Schweitzer, Wittmeyer & Horner, 2005). This discovery suggested: first, that it may be possible to assess gender and sexual maturity in fossil dinosaurs and birds using histology to demonstrate the presence of MB; second, that this reproductive tissue, previously limited to birds, may have evolved prior to the origin of birds, in a more inclusive clade of theropod dinosaurs; third, that the presence of this tissue sheds light on reproductive age in extinct theropods; and fourth, that theropod dinosaurs including birds may have shared at least some elements of reproductive strategies to the exclusion of other archosaurs. Because extant crocodilians cannot form MB (Elsey & Wink, 1986; Schweitzer et al., 2007), the presence of this tissue in a non-avian theropod dinosaur suggested its origin in this lineage prior to the divergence of Aves.

Since this first identification, MB has also been reported in several other non-avian dinosaurs, including taxa outside the theropod lineage (Lee & Werning, 2008; Hübner, 2012), as well as pterosaurs (Prondvai & Stein, 2014; Prondvai, 2017). It has also been reported in two Mesozoic bird lineages recovered from the Jehol biota: in *Confuciusornis* (Chinsamy et al., 2013) and in two enantiornithines (an enantiornithine indet., O’Connor et al., 2018 and in *Avimaia schweitzerae*, Bailleul et al., 2019). Definitive evidence in support of this identification in *Avimaia* is provided through the preservation of an intra-abdominal egg, indicating this adaptation evolved outside the crown clade (Bailleul et al., 2019). However, because some pathologies can produce MB-like tissues in extant birds (Canoville, Schweitzer & Zanno, 2019), it has been noted that unambiguous identification of fossil tissues as reproductive MB requires more than just morphological similarity, particularly with increasing phylogenetic distance from the avian lineage, and/or when it appears in patterns different from those seen in extant birds (O’Connor et al., 2018). Although chemical and antibody staining of MB tissues in *T. rex* support the original diagnosis, given that the fossil tissues reacted to these methods in a manner similar to birds, later studies also shown that at least one pathology is also chemically consistent with MB (Canoville et al., 2018). We propose that multiple lines of evidence taken together are required to support the identification of MB in fossil taxa, including (but not limited to) skeleton-wide distribution (i.e., more than one element), absence of indicators of pathology, and histological consistency.

In summary, the evolutionary origin of MB is still the subject of debate: it is either considered to have evolved prior to the origin of Coelurosauria (Schweitzer, Wittmeyer & Horner, 2005; 2016; Canoville, Schweitzer & Zanno, 2019) or earlier, outside of Dinosauria (i.e., also hypothesized to be present in pterosaurs; Prondvai & Stein, 2014; Prondvai, 2017). A reanalysis of the potential MB reported in the skull of pterosaurs must be made, as it does not appear to meet any criteria of consistency with extant MB; i.e., it was reported in juveniles as well as adult specimens; it was reported in the mandibular symphysis but not elsewhere in the body (a pattern not observed in any living birds; Canoville, Schweitzer & Zanno, 2019); and the endosteal origin of the purported MB is unclear.

Further histochemical studies on purported fossil MB may clarify this issue in the future (O’Connor et al., 2018, based on the chemical results of Canoville et al., 2018). It is clear that robust conclusions regarding the presence of MB in fossils can no longer rely solely on standard paleohistology and must involve other methods to rule out pathology and or identify a chemical signal unique to MB (e.g., see the immunological test that ruled out osteopetrosis; Fig. 5 in Schweitzer et al., 2016).

**Figure 5:**
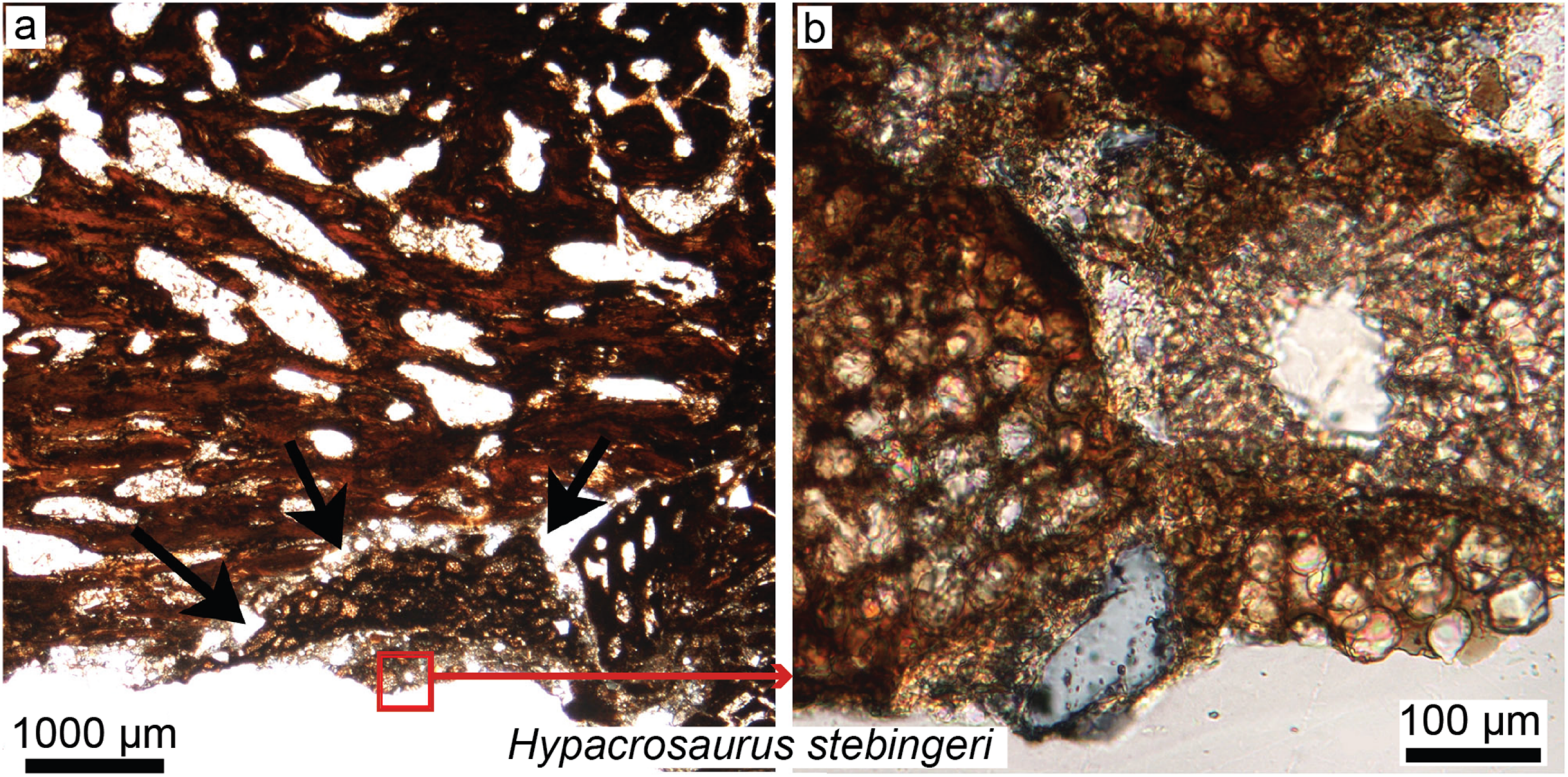
Petrographic ground sections in the maxilla of the duck-billed dinosaur *Hypacrosaurus stebingeri* (MOR 548, a nestling) showing the first evidence of “avian” secondary cartilage (SC) in a non-avian dinosaur. (A) A nodule of SC (black arrows) is found nearing the more internal maxillary bone. (B) A close-up shows hypertrophic chondrocyte lacunae, typical of calcified cartilage. This nodule is found between the maxilla and the coronoid process of the dentary. Although not preserved here, some hyaline cartilage was certainly present as well, in continuity with the calcified cartilage. The cartilage may have facilitated the “rubbing” of the maxilla and coronoid process of the dentary during mastication, as cartilage has shock-absorbing, cushioning properties. It also suggests that some movement was possible at this joint (although the joint structure, or the exact amount of movement, is not clear at this point). Secondary cartilage is also found on the skull bones of living birds, and its discovery in an ornithischian dinosaur suggested that birds inherited this tissue from their non-avian dinosaur ancestors. Modified from Bailleul, Hall & Horner (2012).

## XXIst century trends: skull histology

### Dinosaur skull histology and growth

Paleohistological studies beginning in the 1970s focused almost entirely on features preserved in postcranial bones. This is because limb bones are considered the most useful elements for skeletochronology and studying growth (Horner, De Ricqlès & Padian, 1999; Sander, 2000a; Chinsamy, 2005; with dorsal ribs as sometimes better alternatives; Erickson et al., 2004; Waskow & Sander, 2014; Waskow & Mateus, 2017), but also because features preserved in cranial elements are considered to be more phylogenetically informative than those of the postcrania, hence destructive analyses are discouraged.

**Figure 4:**
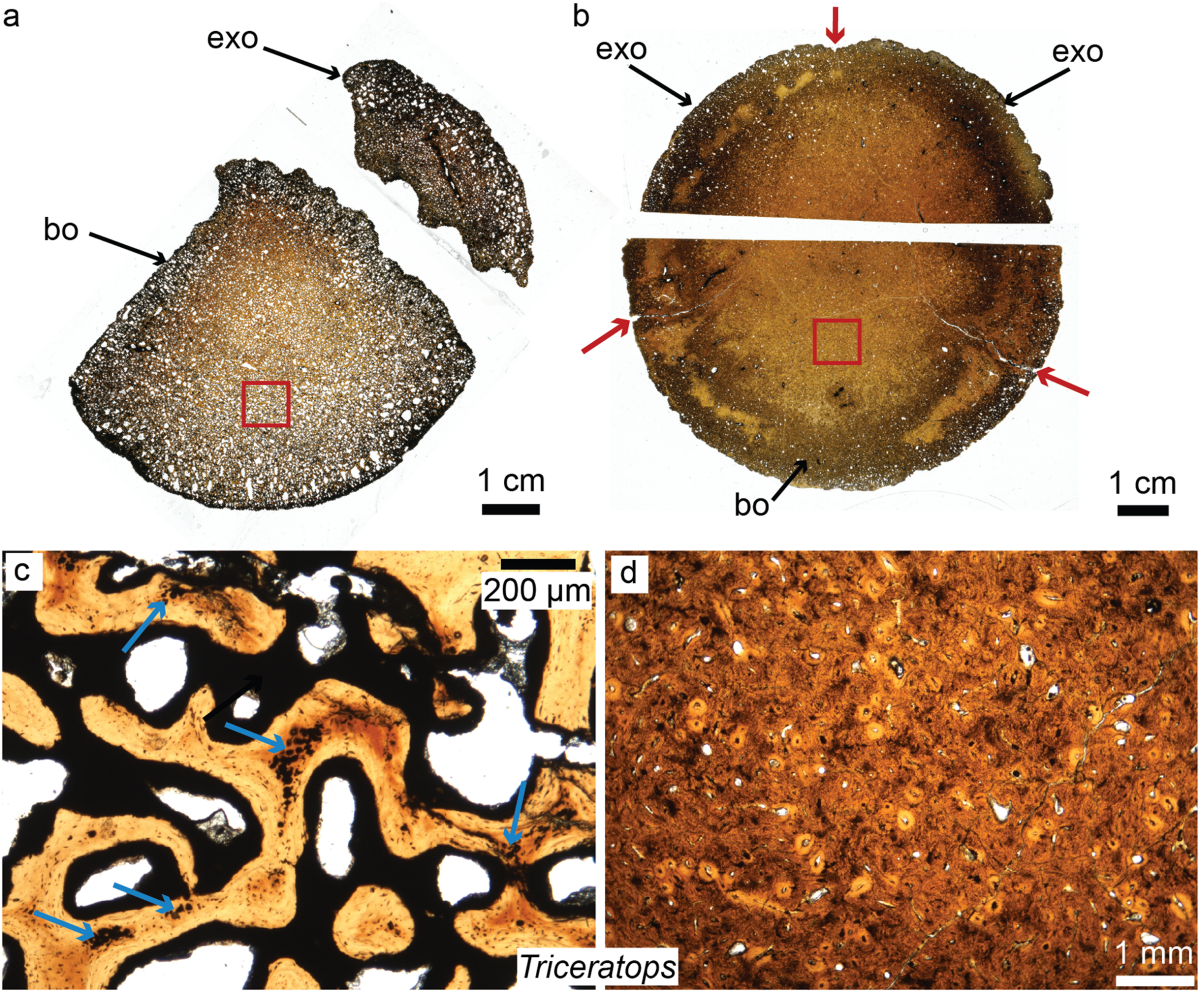
Petrographic ground sections of the occipital condyles of two young *Triceratops*, MOR 1110 (A–C) and MOR 8657 (B–D). (A) ****The first occipital condyle is still unfused (composed of a basioccipital (bo) and two exocipitals (exo) on each side, but only the right one is shown in (A). Magnification of the red square in (A) is shown in (C) and shows the condyle is composed of a highly vascularized, cancellous bone. Calcified cartilage islands can be seen within bony trabeculae (blue arrows). (B) The older occipital condyle (B) is fully fused. Magnification of the red square in (B) is shown in (D). It has a much less vascularized, and more compact bone (D). Sectioning the same cranial element in multiple ontogenetic stages can reveal unknown aspects of dinosaurian cranial growth. Modified from Bailleul & Horner (2016). ©John Wiley and Sons.

Horner et al. recently pioneered the study of dinosaur cranial tissues, mostly on members of the Marginocephalia (ceratopsians and pachycephalosaurs; e.g., Goodwin & Horner, 2004; Horner & Goodwin, 2009; Scannella & Horner, 2011) partially because postcranial elements are rarely found in association with skull material, and isolated skull material is very abundant for this group. Dinosaurian cranial elements that have been histologically studied include the frill, horns and other cranial elements of ceratopsians, including the iconic *Triceratops* (Scannella & Horner, 2010; Horner & Lamm, 2011; Bailleul & Horner, 2016; see Fig. 4 for the basicranium of *Triceratops*), *Nedoceratops* (Scannella & Horner, 2011), and *Centrosaurus* (Tumarkin-Deratzian et al., 2010); the cranial domes of some pachycephalosaurs (Goodwin & Horner, 2004; Horner & Goodwin, 2009; Bailleul & Horner, 2016); and many isolated dermal and endochondral bones of hadrosaur embryos and hatchlings, including *Hypacrosaurus*, *Maiasaura*, and indeterminate lambeosaurines and hadrosaurines (Bailleul, Hall & Horner, 2012, 2013; Bailleul et al., 2016a). The goal of Horner et al., in examining the histology of a variety of non-avian dinosaurs skull elements, was to shed light on previously unknown aspects of dinosaurian growth and ontogeny undecipherable at the gross morphological scale (e.g., Fig. 4 for basicranial growth in *Triceratops*). For example, comparing the microstructure of *Triceratops* and “*Torosaurus*” frills revealed the possibility that these two taxa represent different ontogenetic stages of a single species (Scannella & Horner, 2010), contradicting long-held diagnoses of these taxa as two separate species. More recently, Horner & Goodwin (2009) and Goodwin & Evans (2016) came to the same conclusion regarding three (previously distinct) species of pachycephalosaurs (*Pachycephalosaurus*, “*Stygimoloch*” and “*Dracorex*”) found in the same deposits, introducing the term “ontogimorph” to describe different ontogenetic stages belonging to members of a single species. These studies have demonstrated the importance of cranial histology for making accurate assessments of species diversity through time in clades with elaborate cranial ornaments.

Studies describing the cranial histology of extant archosaurs with the main goal of clarifying inferences regarding cranial ontogeny in fossils are rare. Growth at sutures (i.e., the fibrous articulations linking most dermal bones) and synchondroses (i.e., the cartilaginous joints linking basicranial endochondral bones) has been investigated in ontogenetic series of American alligators and emus, and compared to some extinct dinosaurs and crocodiles (Bailleul et al., 2016b; Bailleul & Horner, 2016). These studies concluded that extreme caution should be taken when trying to infer ontogeny in fossils using sutures alone. However, to fully understand cranial growth in dinosaurs, more widespread investigations of living archosaurs are necessary. So far, no extant analogs have been identified that may be used as suitable proxies for understanding dinosaurian skull growth and microstructure. Many non-avian dinosaurs possessed extensive and often “bizarre” osseous cranial ornaments and displays (Goodwin & Horner, 2004; Padian & Horner, 2011; Farke et al., 2013) that are not found in extant crocodiles, nor to the same extent in living birds (i.e., hornbills and cassowaries do have some cranial ornamentation, but most identifying features are soft tissue rather than bony elements).

### Other new potential areas of investigation for dinosaur skull histology

Although Enlow & Brown (1957) sectioned dinosaur skull material more than 50 years ago (also see Coombs, 1971), comprehensive study of dinosaurian skull histology is a fairly new trend, and its full potential has yet to be explored. Many non-growth-related paleobiological questions could be addressed through microscopic examination of cranial elements. Notably, here we give three brief examples in the following sections:

#### Histological correlates of cranial muscle attachments

Histological correlates of muscle insertions have been described in the heads of extant birds (Hieronymus, 2006), as well as facial epidermal structures in ceratopsids (Hieronymus et al., 2009; Tumarkin-Deratzian et al., 2010), in the attempt to reconstruct dinosaurian cranial soft-tissues. Even though reconstructing soft-tissues in dinosaurs using histological correlates has limitations (notably because not all soft-tissue insertions leave marks directly on the cranial bones (Hems & Tillmann, 2000; Hieronymus, 2006, also see Discussion in Petermann & Sander, 2013 and Lambertz, Bertozzo & Sander, 2018) further investigation in combination with other methods will surely improve reconstructions of dinosaurian cranial anatomy.

#### Timing of evolution of dinosaurian cranial tissues and “avian” secondary cartilage

Secondary cartilage, a presumably exclusively avian tissue (Hall, 1967, 1968, 2000), has been discovered in the skulls and cranial joints of some ornithischian embryos and nestlings (Fig. 5; Bailleul, Hall & Horner, 2012, 2013). The skull of vertebrates is composed of two main types of bones: membrane bones that arise directly within the mesenchyme as bone blastemas, and endochondral bones, which first arise as primary cartilage models before being (fully or partially) replaced by bone (Hall, 2005; Couly, Coltey & Le Douarin, 1993). To avoid any confusion, we will refer to these two types of “bones” as “membranous elements” and “endochondral elements.” In modern birds, secondary cartilage is found exclusively on membranous elements, either as articular cartilage (e.g., in ducks, secondary cartilage is found on the squamosal, within the socket that articulates with the quadrate; Bailleul, Witmer & Holliday, 2017) or at muscle or ligamentous insertions (e.g., on the surangular underlying the *ligamentum squamosomandibulare* in the chick; Hall, 1968). Note that mammals also have secondary cartilage, but based on parsimony and different mechanisms of tissue initiation, it has been determined that avian and mammalian secondary cartilages are not homologous and arose independently during evolution (i.e., in birds, mechanical stimulation is required for both the initiation and maintenance of secondary cartilage, whereas in mammals it is only required for its maintenance; Hall, 2000; also see Supplemental Material in Bailleul, Hall & Horner, 2012).

Avian secondary cartilage arises from the periosteum of membranous elements, and in comparison, the articular cartilage found on the ends of endochondral elements (e.g., the quadrate, and elements of the chondrocranium) is a type of primary cartilage ( because it originated from the primary cartilage model; Hall, 1967, 1968, 2000; Bailleul, Hall & Horner, 2012). Secondary cartilage may differ slightly microstructurally from the more common primary cartilage (i.e., it may have less extracellular matrix, at least very early in ontogeny, or it may be more fibrous later in ontogeny, e.g., see Bailleul, Hall & Horner, 2012; Bailleul, Witmer & Holliday, 2017) but the identification of this tissue is mostly based on its location (i.e., on a membranous element) and not purely on histological differences (Bailleul, Hall & Horner, 2012). The presence of this “avian” tissue in non-avian dinosaurs suggests that birds inherited this tissue from their non-avian dinosaur ancestors (Bailleul, Hall & Horner, 2012), and provides further phylogenetic evidence at the microscopic scale for the close evolutionary relationship of these groups. Together with MB (Schweitzer, Wittmeyer & Horner, 2005; Schweitzer et al., 2016), these could be considered the only two skeletal tissues (Figs. 3 and 5) shared exclusively between dinosaurs and birds (being absent in crocodilians, and other more basal archosaurs), thus supporting the dinosaurian origin of birds.

#### Skeletal tissues and cranial biomechanics

Skeletal tissues can shed light on cranial biomechanics and function. Although some functional aspects of the postcranium in both fossil and extant archosaurs have been investigated using histology (De Ricqlès et al., 2000; Ponton et al., 2007; Zhao et al., 2013; Cubo et al., 2015; Tsai & Holliday, 2015; Mitchell et al., 2017), in comparison, very few functional histological studies on cranial elements exist. Histological analyses of cranial skeletal tissues conducted to reconstruct skull biomechanics are still in their infancy, but they have the potential to be highly informative. Specifically, secondary cartilage, in addition to its phylogenetic importance, has a biomechanical significance. Experimental studies that induce cranial joint paralysis and inhibit embryonic motility (e.g., movements such as beak clapping), creating an immobile environment in developing birds, show that this tissue does not develop in the absence of movement, whereas primary cartilage forms in both mobile and immobile conditions (Murray & Drachman, 1969; Persson, 1983; Hall, 1972). Therefore, the presence of secondary cartilage within a fossil joint (Fig. 5) can be used to infer mobility. The hypothesis that physical movement may be responsible for secondary cartilage formation has been previously proposed for some cranial joints in both birds and mammals (Murray, 1963; Murray & Drachman, 1969; Bareggi et al., 1994), but the degree and/or direction of such movement has not been quantified. Nevertheless, cranial tissues have great potential for more accurate inferences regarding skull structure and function (e.g., cranial kinesis or akinesis) in non-avian dinosaurs and other fossil archosaurs (e.g., see Bailleul & Holliday, 2017; Lessner et al., 2019).

Future histological studies on the skulls of extant archosaurs will expand our ability to reconstruct cranial biomechanics in their extinct relatives (especially studies that would also provide quantitative data), and will, no doubt, elucidate other aspects of the evolution and function of extinct dinosaurs and birds.

### Other dinosaurian tissues

The histological study of other non-limb postcranial elements has also become increasingly common in archosaur paleobiology over the last decade, and it was noted that they share similarities with many dinosaurian cranial tissues. These elements include the osteoderms of extant alligators (Dacke et al., 2015), basal archosauriformes (Scheyer & Sander, 2004; Scheyer, Desojo & Cerda, 2014), and non-avian dinosaurs such as ankylosaurs (Vickaryous, Russell & Currie, 2001; Scheyer & Sander, 2004) and sauropods (Cerda & Powell, 2010; Rogers et al., 2011); the osteoderms and plates of stegosaurs (Scheyer & Sander, 2004; Main et al., 2005; Hayashi et al., 2012); the tail clubs of ankylosaurs (Hayashi et al., 2010); and the ossified tendons of hadrosaurs (Adams & Organ, 2005; Horner, Woodward & Bailleul, 2016). From these studies it is clear that non-limb bone postcranial elements may not present “standard” limb bone histology in that they lack the three “standard” bone tissues: woven, parallel-fibered, or lamellar (Prondvai et al., 2014), deposited by periosteal ossification (Fig. 1). Instead, many of these tissues are extremely fibrous and it is often impossible to identify the clear, stellate osteocyte lacunae typically found in the cortical bone of limb elements. This “fibrous” microstructure has also been observed in many of the dinosaurian cranial ornaments mentioned previously (Horner & Lamm, 2011; Goodwin & Horner, 2004). To our knowledge, some of these fibrous tissues (notably, many of those in pachycephalosaur skulls) have no extant analogs, making it difficult to infer their mode of formation, function, or ontogenetic origin.

**Figure 6:**
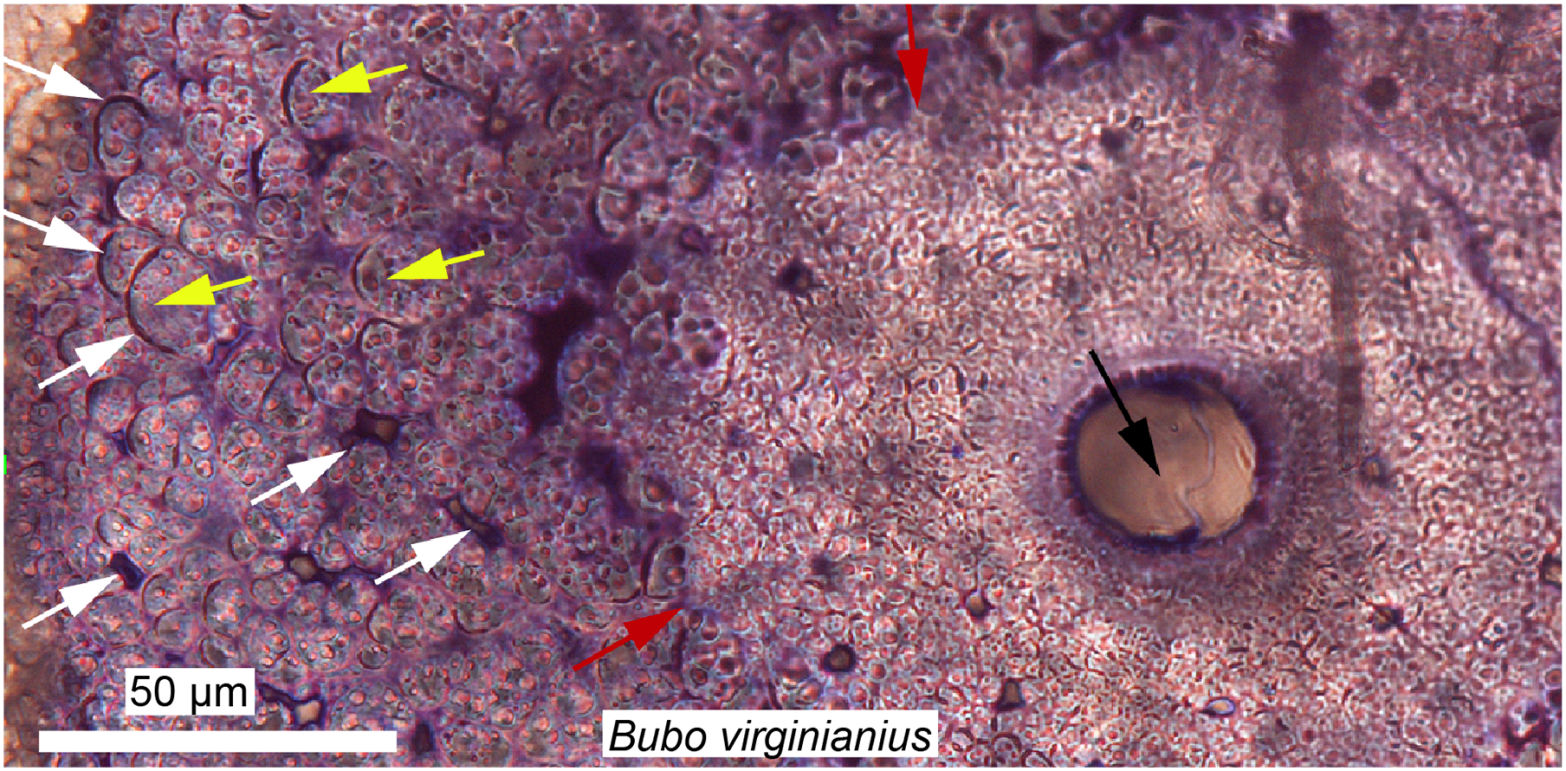
Ground section of a mineralized tendon from *M. extensor carpi radialis* of *Bubo virginianus* (Great Horned Owl), showing that it does not present the microstructure typically expected for bone. The tissue is made of small collagen fiber bundles and fascicles (yellow arrows), separated from each other by arc-shaped spaces (white arrows). Red arrows are pointing at an irregular border between two types of tissues and probable unmineralized fiber fasciles. No regular bone cell lacunae (with an elongated morphology and canaliculi) can be seen anywhere in these sections, and this reflects a unique mode of skeletal tissue formation, different from that of typical bone. Many dinosaurian and archosaurian tissues have been found with a similar microstructure. Modified from Bailleul & Horner (2016).

These fibrous tissues are generally referred to as “metaplastic bone,” a term first used by Haines & Mohuiddin (1968) to describe *a skeletal tissue formed in the absence of osteoblasts through a direct transformation of one tissue type to another*. This definition is confusing, because metaplasia can only be properly defined at the cellular level (i.e., with the transformation of one cell population into another), but not at the histological level. For example, the initial formation of osteoderms in the American alligator occurs with neither a condensation of periosteum nor osteoblasts, but rather via direct mineralization of pre-existing connective tissues within the dermis (Vickaryous & Hall, 2008). The cells that first deposit this “bone” (or mineralized tissue) are still unknown, but it is suggested that a population of fibroblasts is responsible (i.e., fibroblasts that alter their extracellular secretions to deposit bone matrix, and later on acquire an osteoblastic phenotype; Vickaryous & Hall, 2008). A similar process has been described in the “ossified” tendons of extant birds (Fig. 6; Landis & Silver, 2002; Adams & Organ, 2005).

Using the histological characters of some tissues referred to as “metaplastic” in extant archosaurs (Fig. 6), Horner, Woodward & Bailleul (2016) analyzed and compared various cranial and non-limb postcranial elements of non-avian dinosaurs, and hypothesized that these tissues all formed via similar metaplastic processes. The results of this study suggest that, indeed, many ornamental bones (cranial and postcranial) in non-avian dinosaurs may not be formed via the normal periosteal ossification processes present in other vertebrates. Similarly, Cerda (2009) showed that some elongated slender bones, most likely sauropod cervical ribs, were in fact ossified tendons. A few years later Klein, Christian & Sander (2012) confirmed this by sampling unquestionable sauropod cervical ribs and showed that only the anterior and posterior processes are ossified tendons (but the core, capitulum, and tuberculum are true cervical ribs). These structures in sauropods are also strikingly similar to the prezygapophyseal rods of *Deinonychus* (Horner, Woodward & Bailleul, 2016).

A broader sampling of extant tissues, both within and outside Archosauria (e.g., see Scheyer et al., 2007 for a study on chelonians; Wolf, Kalthoff & Sander, 2012 and Heck et al., 2019 for mammals) are necessary to fully understand and properly recognize true metaplastic tissues in fossils. We argue that although it may be well supported in some cases, there is no unequivocal and direct evidence that a fossilized tissue (observed in a ground section) formed by cellular metaplasia. The cellular populations responsible for the formation of metaplastic tissues in extant species is still unclear (e.g., recent histological research suggest the initiation of osteoderm formation in American alligators comes from endothelial cells, rather than fibroblasts; Dubansky & Dubansky, 2018); and a definitive correlation of cellular metaplasia with a specific tissue type (observable in ground section) has not been made. This lack of understanding of “metaplastic” tissues in extant and fossil species comes from the fact that it is difficult to follow the transformation of a cellular population into another using standard ground (or paraffin) sections. Instead, cellular labeling (e.g., using fluorescence and immunohistochemistry) must be employed. Neontological studies are necessary to shed light on the seemingly unfamiliar, metaplastic-like fibrous tissues that are observed throughout the skeleton of non-avian dinosaurs.

### Dinosaur oral histology

The dental histology of extant archosaurs (limited to crocodilians because all extant birds are edentulous) is well documented (Kvam, 1960; Westergaard & Ferguson, 1990; Tadokoro et al., 1998; McIntosh et al., 2002). Crocodilian teeth and the bone tissues of the upper and lower jaws that supports them involve four main mineralized tissues: enamel, dentine, cementum, and alveolar bone (Gaengler, 2000). Unlike alveolar bone and cementum, which contain osteoblasts and cementoblasts, respectively, enamel and dentine are mostly acellular (dentine only contains cellular extensions called odontoblasts processes; and enamel is completely acellular and hypermineralized; Hall, 2005). The teeth of crocodiles such as *Alligator mississippiensis* and *Caiman sclerops* differ from those of other extant reptiles in that they are not ankylosed into the jaws; instead they are attached to alveolar bone via a periodontal ligament (Kvam, 1960; Gaengler, 2000; McIntosh et al., 2002). This condition is similar to that of mammals, and has also recently been described in non-avian dinosaurs and toothed stem birds (Leblanc et al., 2016; Dumont et al., 2016). Some of the earliest studies of dinosaur tooth histology focused on dentine, which is deposited daily (Erickson, 1996a, 1996b). Erickson used histological data to infer dinosaurian rates of tooth development and replacement using marks referred to as Von Ebner’s lines which form daily with the deposition of dentine in crocodilians (and other extant groups) (Erickson, 1996a, 1996b). Erickson reasoned that the rate of tooth replacement could be calculated by counting the number of incremental lines in a functional tooth and subtracting the number in the successive replacement tooth. These studies demonstrated that for any given tooth size, the teeth of theropod dinosaurs formed and grew at much slower rates than those of herbivorous (e.g., ceratopsians and hadrosaurid) dinosaurs (Erickson, 1996a). This is undoubtedly linked to the evolution of the dental battery in ceratopsians and hadrosaurids (Leblanc et al., 2016). More recently, microscopic analyses have provided new data that further elucidate the formation of dinosaurian teeth, such as hadrosaurs (Erickson et al., 2012, 2017; Leblanc et al., 2016), *Triceratops* (Erickson et al., 2015), carnivorous theropods (Brink et al., 2015), herbivorous theropods (i.e., therizinosaurs, Button et al., 2017), sauropods (García & Zurriaguz, 2016), and fossil birds (Dumont et al., 2016), although this list is not exhaustive.

Some earlier studies focused on the evolution and microstructure of enamel (Sander, 1999, 2000b). Unlike in mammals, enamel in non-mammalian amniotes (including archosaurs) lacks prisms (i.e., tightly packed and organized mass of hydroxyapatite crystals). This tissue cannot be easily studied using standard ground-sections and the best way to investigate enamel is via scanning electron microscopy (SEM) (Koenigswald & Sander, 1997; Sander, 2000b).

Sander (1999) analyzed a wide sample of extant and extinct sauropsids (including squamates, crocodilians, birds, saurischian, and ornithischian dinosaurs) and found that tooth enamel shows great structural variety and diversity. He found that the major factor determining these microstructural differences was not phylogeny, but rather the surface morphology of the teeth (e.g., with or without ridges on the surface; Sander, 1999). A more recent analysis including a greater diversity of dinosaur taxa suggested that the three-dimensional arrangement of enamel types and features within a tooth (i.e., the schmelzmuster, not the enamel microstructure complexity) is most useful for diagnosing some dinosaur clades (Hwang, 2005). Sander (1999) also noted that among all of the dinosaurian taxa he analyzed, only derived ornithopods (hadrosaurids) had a unique enamel type (i.e., wavy enamel) which he considered a synapomorphy. Wavy enamel (in hadrosaurs) is associated with a specific surface micromorphology, very similar to that seen in ceratopsians, and Sander (1999) hypothesized that this micromorphology may have been linked to the biomechanics of the complex dental batteries found in these two groups

**Figure 7:**
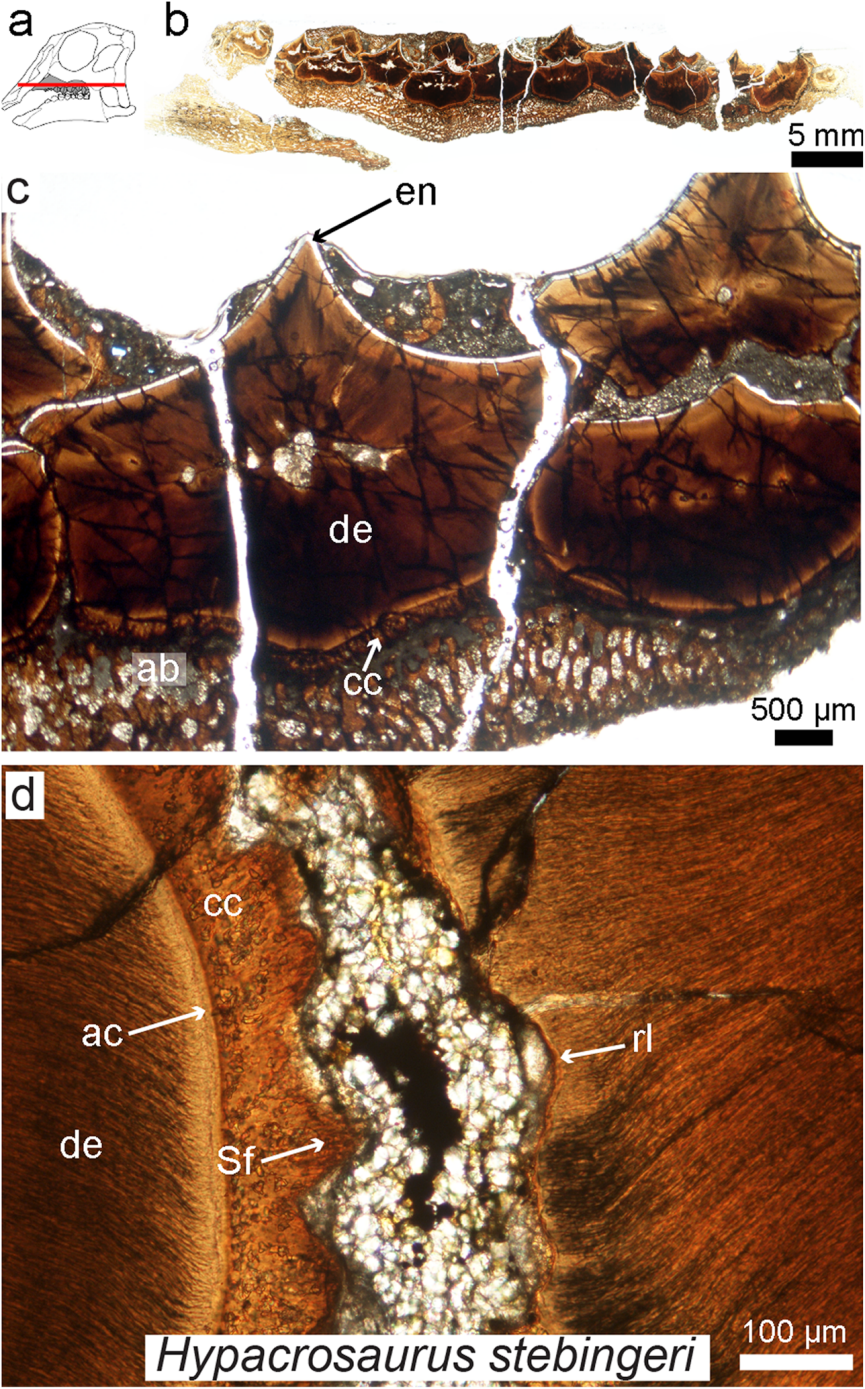
Petrographic ground section of an isolated maxilla of a *Hypacrosaurus* embryo (MOR 559). (A) Schematic representation of the skull of a *Hypacrosaurus* embryo with orientation of the cut on the maxilla. (B) Whole-view image of a transverse section through the maxillary battery near the occlusal surface. (C) A close-up image in the same transverse section shows different dental tissues (alveolar bone, ab; enamel, en; dentine; de; cellular cementun, cc). (D) Higher magnification shows that teeth contacted each other via soft tissues (i.e., periodontal ligament), as reflected by Sharpey’s fibers (Sf) within the cellular cementum and the space between the teeth filled with minerals and sediment. Therefore, in addition to possessing a ligamentous attachment with alveolar bone, hadrosaurs had a unique tooth-to-tooth fibrous attachments in which each individual tooth within the battery was suspended to its neighbors through soft tissue connections. Other abbreviations: ac, acellular cementum; rl, resorption line; Modified from Leblanc et al. (2016).

Today the hadrosaurian dental battery is regarded as one of the most complex oral adaptations ever to have evolved, with nothing even remotely comparable to this complex system present in extant vertebrates (Fig. 7). Indeed, some derived hadrosaurs had up to 300 teeth in each jaw ramus forming an intricate grinding system capable of efficiently processing plant material. Erickson et al. (2012) created three-dimensional biomechanical wear models based on the different material properties of the involved tissue types and wear patterns observed in hadrosaur teeth, revealing a surprisingly complex system of tooth wear and replacement. More recently, Leblanc et al. (2016) attempted to reconstruct hadrosaur dental ontogeny by looking at a growth series, from embryo (Fig. 7) to adult, and proposed that in addition to possessing a ligamentous tooth attachment between the teeth and alveolar bone, these dinosaurs had unique tooth-to-tooth fibrous attachments in which each individual tooth within the battery was attached to its neighbors through soft tissue connections (Fig. 7). This study also revealed that the teeth of hadrosaurs were not shed as previously assumed (Edmund, 1960), but that they were in fact ground down and resorbed completely. Bramble et al. (2017) demonstrated how gradual tooth migrations and movements were controlled via the periodontal ligament within the dental battery.

While enamel microstructure (using SEM) has been studied across a wide range of dinosaur taxa (e.g., see the latest review by Hwang, 2011), this is not the case for other dental tissues. The histology of dentine, cementum, or alvelovar bone (as well as indicators of the periodontal ligament) must be further investigated in other groups of dinosaurs, and will most likely reveal further complexity in dinosaurian oral tissues. Such studies need not be destructive; recently it has been shown that synchrotron technology (i.e., “virtual paleohistology”; Sanchez et al., 2012) can be used to visualize histological features in fossil bird teeth (Dumont et al., 2016). New scanning technologies combined with standard ground-sections provide exciting new possibilities for the future of oral paleohistology, and the field as a whole.

## Molecular paleontology: a new way to study the tissues of dinosaurs?

This review has focused thus far on traditional paleohistology, which uses petrographic ground sections to visualize mineralized fossil tissues (e.g., bone, dental tissues, calcified cartilage, the tissues in ossified tendons) and the cells that secreted them. However, molecular paleontology is also included here because this sub-discipline of paleontology offers an even deeper understanding of the biology of fossil tissues, and thus can be considered a form of paleohistology. The first molecular studies on fossil archosaurs were those of Pawlicki, Korbel & Kubiak (1966), Pawlicki (1977), Pawlicki & Nowogrodzka-Zagórska, 1998), and were followed by those of Schweitzer et al. in the late 1990s (Hedges et al., 1995; Schweitzer et al., 1997a, 1997b, 1999a, 1999b; Schweitzer & Horner, 1999). The development of molecular paleontology has not been as straightforward as that of the field of paleohistology, most likely due to two untested yet widespread assumptions: (1) that fossils do not retain any original organics, but rather these are completely replaced; and (2) that if some biomolecules do indeed survive into the rock record, a very short temporal range is predicted for their survival, e.g., ~1 million years for proteins, and ~100,000 years for DNA (Lindahl, 1993; Willerslev & Cooper, 2005, but see Orlando et al., 2011). However, over the last decade, these assumptions regarding the preservation and longevity of organic remains have been challenged by the discovery of fossilized remnants of a plethora of organic materials, such as entire soft tissue structures (e.g., blood vessels found within bone; Schweitzer et al., 2005a; Schweitzer, Wittmeyer & Horner, 2007), cellular structures (e.g., bone cells, Cadena & Schweitzer, 2012; Schweitzer et al., 2013), extracellular proteins (e.g., collagen found in the extracellular matrix of bone, Schweitzer, Wittmeyer & Horner, 2007; Lindgren et al., 2011; eggshell proteins in dinosaur eggs, Schweitzer et al., 2005b), intracellular proteins (e.g., heme compounds, Schweitzer et al., 1997b; or actin, Schweitzer et al., 2013), and pigments, like melanin (Vinther et al., 2008; Lindgren et al., 2015, 2017) or the protoporphyrins found in dinosaur eggshells (Wiemann, Yang & Norell, 2018).

**Figure 8:**
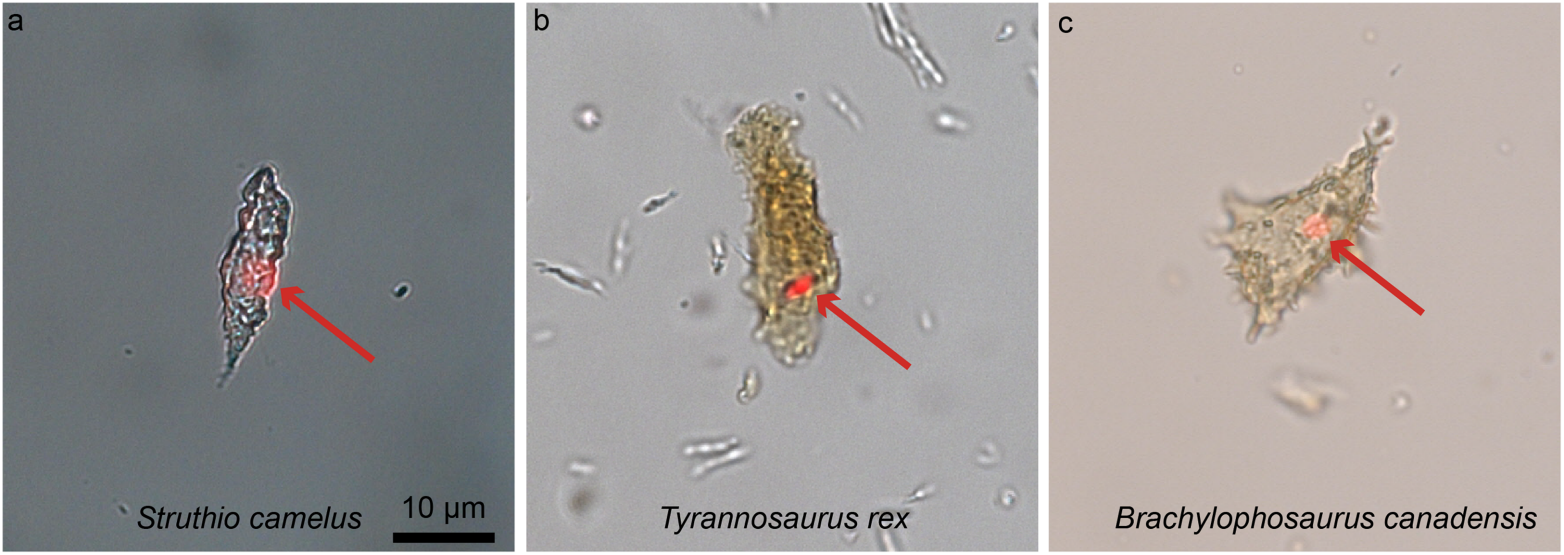
Ostrich (A) and dinosaur (B–C) cellular response to the DNA intercalating dye propidium iodine (PI). Extant ostrich osteocytes (A), isolated osteocytes from the extinct theropod *T. rex* (B), and osteocytes from the hadrosaur *B. canadensis* (C) show identical response to PI (red arrows, consistent with the location of cell nuclei). This strongly suggests a compound chemically consistent with DNA, can survive tens of millions of years. PI requires double-stranded DNA to react, and only stains the nucleus of dead cells; these data support the presence of a compound with these characteristics. The data are not consistent with binding that occurs in bacteria, which are orders of magnitude smaller. However, although this binding pattern is consistent with that seen in extant samples, only sequence data can fully confirm the origin of this material (see Schweitzer et al., 2013 for additional data). Images are at the same scale.

One of the most unexpected biomolecular discoveries (Fig. 8) was the identification of intracellular compounds inside dinosaur bone cells (between 78 and 66 million years old), determined to be chemically consistent with DNA using multiple and independent lines of evidence (i.e., in *Brachylophosaurus canadensis* and *T. rex*; Schweitzer et al., 2013; Fig. 8). The endogeneity of these structures is supported by histochemical and immunological techniques that require demineralization. However, other less invasive alternative methods involving spectroscopy (e.g., Raman, or FTIR) can also be used as fairly non-specific means to test for molecule preservation in the fossil record (e.g., Wogelius et al., 2011; Reisz et al., 2013; Bertazzo et al., 2015; also see review in Pan, Hu & Zhao, 2018 for all the techniques available). In fact, these two types of methods, traditional and spectral, are complementary and should be used in tandem when possible (Lindgren et al., 2018). The ultimate validation of the origin of molecular fragments whether endogenous or exogenous, is sequence data. However, given that comparative databases are still expanding, and sequences from many proteins and taxa are being added to these databases, once-unique sequences have been identified (Buckley et al., 2017), and will continue to be identified in living organisms, a “dinosaur-specific” sequence is not sufficient to support endogeneity. Sequence data, in conjunction with in situ studies are required.

Recently, the hypothesis that iron is somehow involved in inter- and intramolecular polymerization and may facilitate the preservation of biomolecules (Schweitzer et al., 2014) was supported through the use of Raman microspectroscopy, which demonstrated the presence of molecular cross links in preserved soft tissues recovered from fossil bone (Wiemann et al., 2018). Wiemann et al., suggest that original proteins were transformed by polymerization resulting from the formation of inter- and intramolecular crosslinks. Furthermore, these authors proposed that to promote crosslink formation, an oxidative environment was required (e.g., sediments with grains with a large surface area, or perhaps iron-rich sandstones). This may be problematic, because the immediate environment of degrading organics is primarily reducing, as microbial participants of degradation utilize oxygen. Thus, a reducing environment would presumably dominate deep in bone tissues until all superficial soft tissues were destroyed. However, these data suggest a mechanism that may result in the preservation of cellular or tissue structures and the proteins comprising them that must be further tested. This recent study adds to existing data that links soft-tissue preservation to aspects of the burial environment, providing valuable information to guide future molecular studies (Wiemann et al., 2018).

Molecular data have revolutionized the way we study living organisms, shedding new light on phylogeny, evolutionary strategies, co-evolution of pathogens, and physiological responses, to name a few. Similarly, these recent molecular findings from fossil archosaur bones are slowly revolutionizing the way that the community perceives both biomolecule and tissue preservation in deep time. Original molecular signal is closely linked to exceptional histological preservation (Hagelberg et al., 1991; Haynes et al., 2002; Hedges, 2002), and overall, histological integrity influences and correlates with molecular studies, by supporting a lack of diagenetic alteration that would most certainly affect molecular preservation. Because of this correlation between molecular and histological preservation, paleohistology plays a vital role in molecular studies of fossils and serves as an important screening tool in selecting fossils appropriate for molecular studies.

**Figure 9:**
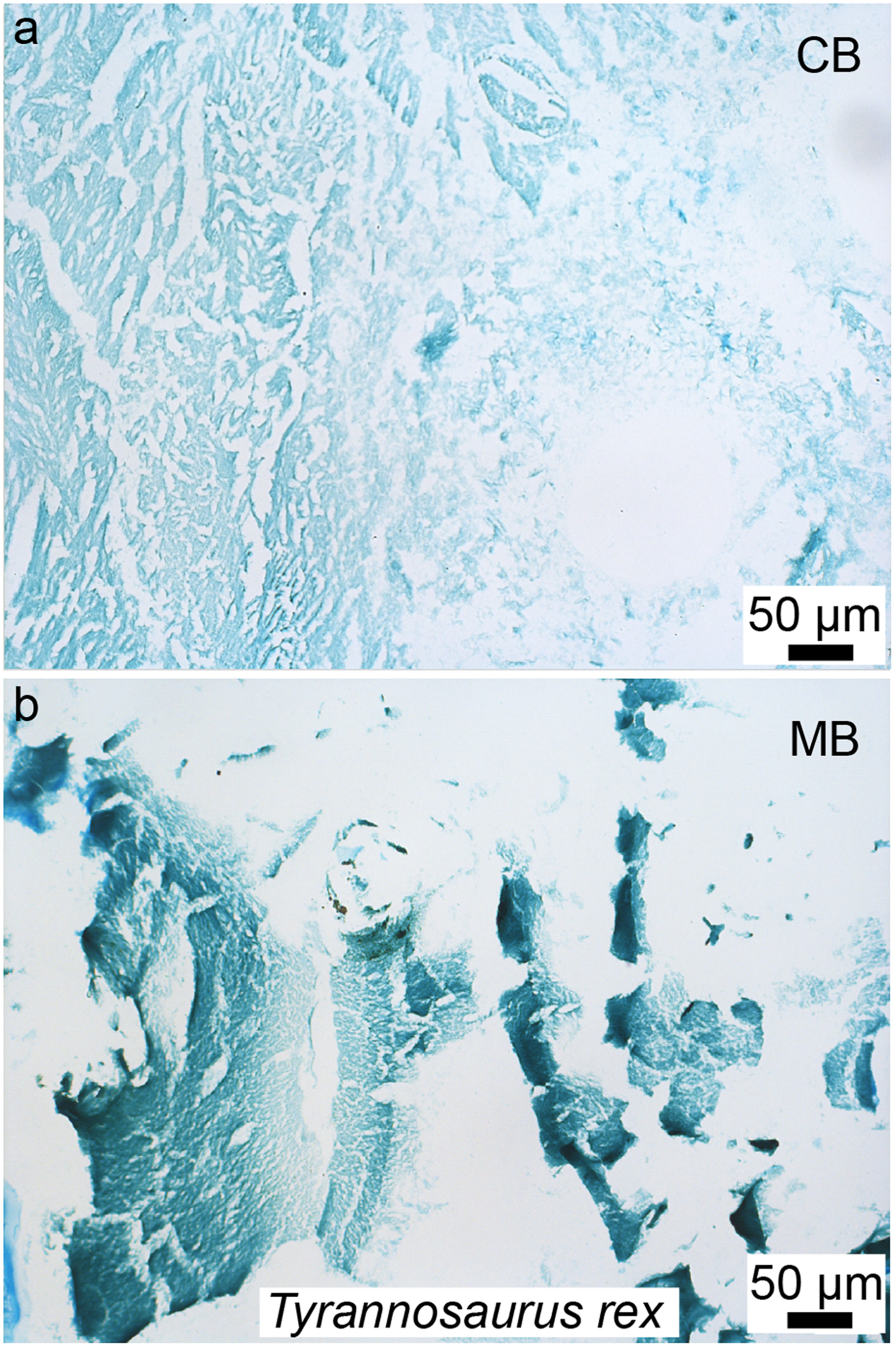
Paraffin thin-sections paired with alcian blue histochemical staining of demineralized cortical bone (A) and medullary bone (B) of *T. rex* (MOR 1125). This stain capitalizes on the differential presence of sulfated glycosaminoglycans found in cortical bone (CB) vs medullary bone (MB), with low amounts in the former (with a faint staining), and a higher amount in the latter (with a more intense staining). The same differential staining pattern is observed in these two tissues in extant birds; which provided additional histochemical similarities between the MB in *T. rex* and the gender-specific, reproductive MB found in extant birds. This new method (paleohistochemistry) can be applied to other fossilized tissues including soft-tissues, and if combined with other microscopic observations and/or techniques, has the potential to revolutionize paleohistology. Modified from Schweitzer et al. (2016).

If paleohistological methods (Lamm, 2013) and molecular methods (Schweitzer et al., 2008) are used in tandem, they have the potential to further elucidate the evolution of fossil tissues at the molecular and/or chemical level, and to shed light on the taphonomic processes resulting in their preservation (Schweitzer et al., 2014; Wiemann et al., 2018). For example, histochemical stains applied to histological sections are routinely used to differentiate extant tissues, based upon differences such as pH (e.g., Alcian blue, or Hematoxylin/eosin) and the presence of aldehyde groups (e.g., Feulgen or PAS stains) or lipid components (e.g., Sudan Black stain). Until very recently, it was thought that chemically staining fossil tissues was impossible, either because of the thickness of undemineralized ground-sections (which are often too dark, masking most underlying tissues), the presence of mineral that obscures chemically differentiated soft tissues, or more commonly, an assumed lack of preservation of the inherent and endogenous biomolecules recognized by such stains. However, using demineralized fossil tissues and embedding them with both water permeable polymers and paraffin wax (routinely used to study extant tissues), Schweitzer et al. (2016) demonstrated that medullary and cortical bone in the iconic *T. rex* were amenable to chemical staining that could capitalize on aspects of the molecules comprising these tissues in a manner similar to tissues from extant organisms (Fig. 9). Additional fossil tissues are currently being subjected to demineralization and staining (data not shown), and we suggest that this new approach holds great potential to gain better identification and understanding of fossil tissues through paleohistochemistry (Fig. 9; for additional staining methods of fossil tissues see Garland, 1989; Zylberberg & Laurin, 2011). Although chemical stains do not have the specificity of other methods (e.g., immunohistochemistry) they can indicate that certain compounds are localized to or sequestered in fossil tissues; that these are consistent with patterns observed in extant controls; and can be used to support the presence of original molecules and/or lack of complete diagenetic alteration of these tissues. However, the lack of specificity limits their usefulness, and chemical staining should always be used together with other analytical tools.

Molecular investigations of paleontological tissues also hold potential to shed light on the origin and composition of still-soft, originally unbiomineralized tissues that is not possible, or not as precise using standard paleohistological ground-sections (but see Chin et al., 2003). In addition to demineralized bone, keratinized tissues, fibrocartilage, eggshell cuticles, and lung tissue, have been examined in dinosaurs and birds (Schweitzer et al., 1999a, 1999b; Moyer, Zheng & Schweitzer, 2016; Pan et al., 2016; Jiang et al., 2017; Wang et al., 2018; Wiemann, Yang & Norell, 2018), but such studies are still rare and, most of the time, controversial. A recent study on a Jurassic ichthyosaur (*Stenopterygius*) employed a comprehensive multidisciplinary experimental approach to demonstrate the exquisite cellular, histological, and molecular preservation of its epidermis, dermis, blubber, and putative liver in this specimen (Lindgren et al., 2018). These analyses almost doubled the evidence for longevity in preserved biomolecules, testifying to the preservation potential of a variety of both biomolecules and tissues, and marking the first application of some analytical techniques to ancient fossils. Such studies show the value and potential of a multi-pronged approach to the study of fossil remains. The histological assessment of these tissues supports physiological inferences, i.e., homeothermy, and crypsis (Lindgren et al., 2018), exemplifying how an understanding soft-tissue microstructure in fossils can be used to answer paleobiological questions with major evolutionary implications.

Any fossils preserving soft tissue remains or evidencing other aspects of exceptional preservation (e.g., beautifully preserved amphibians, McNamara et al., 2009, 2010) would benefit from similar comprehensive analyses at both the molecular and histological levels. The well-preserved birds of the Jehol Biota of Northeastern China present a myriad of soft-tissue structures, such as remnants of ovarian follicles (O’Connor et al., 2013; Zheng et al., 2013), feathers, wing patagium, scales (e.g., Hou et al., 1995; Ji & Ji, 1996; Zhou, Barrett & Hilton, 2003; Pan et al., 2016; Zheng et al., 2017), skin (McNamara et al., 2018), and lungs (Wang et al., 2018), identification of which could be further supported and explored through high resolution paleohistochemistry and/or paleoimmunological techniques.

## Conclusions

In this overview of new methods of investigation applicable to the study of fossil tissues, we: (1) identify general and still unresolved questions that exist in the field of dinosaur paleohistology; (2) review new technical and methodological insights with the potential to provide additional information about the once living animals whose remains we study; and (3) propose new avenues for the field that, together with older, well tested, and well accepted methods, may provide a more complete understanding of the paleobiology of all dinosaurs.

### Some unresolved questions

Paleohistology is a powerful field that has helped us reconstruct many aspects of the biology of extinct dinosaurs and other fossil organisms. Unfortunately, the primary method used in this field is considered too destructive, and its use is still limited largely to clarifying growth-related questions, despite the potential to address a great many other biological questions; histology is only just beginning to reach its potential in paleontology.

Histological studies related to growth and physiology have changed the way that we study non-avian dinosaurs, fossil birds, and other fossil archosaurs, and have expanded our ability to envision their biology, evolutionary relationships, and ontogeny. However, a few questions are not entirely resolved and should still be clarified. Notably, *when did MB (the reproductive tissue found in modern female birds) first appear during evolution* (Schweitzer, Wittmeyer & Horner, 2005; Schweitzer et al., 2016; O’Connor et al., 2018)? This must be resolved before we can fully understand the evolution of the reproductive system in archosaurs. Second, *are all purported LAGs truly annual features?* LAGs have been confirmed as annual markers in many modern mammals (Köhler et al., 2012), but is this the case in modern archosaurs as well? Presumably yes, *however, are there non-annual growth marks that are extremely similar to, and could be easily be mistaken for LAGs in extant archosaurs and in the fossil record?* Even though further work is needed, the very recent study by Woodward (2019) suggests that there is much more to understand about cortical growth marks that previously thought, and this should be further investigated in both fossil and extant archosaurs. *Lastly, do postcranial and cranial growth marks correlate exactly, and if so, how?* Cranial LAGs and other types of growth marks must be further investigated.

Another unresolved question in dinosaur paleohistology concerns “metaplastic bone,” “metaplastic tissues,” or any type of unfamiliar looking tissue. Almost all of our assumptions are based on the mode of formation of avian ossified tendons (Landis & Silver, 2002) and alligator osteoderms, but yet these processes need further clarification in extant species (Vickaryous & Hall, 2008; Dubansky & Dubansky, 2018). It appears that there are a myriad of tissues in the skulls of fossil archosaurs, most notably in pachycephalosaurs, referred to as “metaplastic,” that have not been reported in extant species (Horner, Woodward & Bailleul, 2016; Goodwin & Horner, 2004), hence investigations involving diagnostic techniques that follow entire cell populations (such as immunohistochemistry) are needed to identify and characterize these unusual tissues in modern analogs, if they are present. Secondarily, identification of diagnostic histological characters (observed in ground-sections) must be made to correctly identify fossilized “metaplastic” tissues. We suggest that the term “metaplastic” be used with caution when describing tissues in fossil archosaurs.

### Some methodological and technical insights

Microscopic examination, particularly when paired with molecular methods developed to study extant tissues, can be effective in addressing questions of an evolutionary, or even physiological nature, in ancient fossil tissues. However, their application to the study of ancient fossils is limited, because they are destructive by nature, compromising the integrity of rare and exceptionally preserved material. However, judiciously applied destructive analyses that minimalize damage (e.g., core-drilling; Sander, 2000a; Stein & Sander, 2009) hold enormous potential to recover information regarding the biology of extinct organisms that cannot be obtained through any other means; we encourage more frequent application of methods described herein, when appropriate to the questions being asked, as well as fossil restoration (Lamm, 2013).

Many additional specimens and tissues remain to be studied at the microscopic or molecular level, but most importantly, new techniques must be developed and optimized to account for the unique problems associated with analyses of fossil tissues, to fully attain the potential of paleohistology. Although paleohistological methods are more routinely used than molecular techniques in the field of paleontology, this is changing, and a combined approach employing standard ground sections with standard (e.g., immunohistochemistry on ultrathin sections) or new molecular techniques (e.g., paleohistochemistry on paraffin sections) will revolutionize the field of paleohistology. Combined with non-destructive methods (such as micro-computed tomography, or synchroton technology (Sanchez et al., 2012; Dumont et al., 2009, 2016), these methods can inform on a greater number of fossils and address broader evolutionary questions into deep time.

Non-destructive technologies when they provide histological-grade details, such as virtual histology (Anné et al., 2014; Dumont et al., 2016; Knoll et al., 2018) are of course the ideal methods to observe tissues in paleontology. Non-destructive molecular methods (e.g., some spectroscopic techniques) should also be favored when necessary. However, in reality the relatively limited access to some of these methods and the high costs associated with them (e.g., synchrotron) is still problematic today. The advances of technology will hopefully allow us to significantly reduce the amount of destructive sampling associated with standard paleohistology and molecular paleontology in the near future.

### New avenues: soft-tissues and biomolecules as an integral part of XXIst century paleohistology and paleontology

Most paleohistological and molecular studies on fossil archosaurs have focused on bone because it is the most abundant skeletal tissue that fossilizes. However, many other mineralized biomatrices still remain to be investigated, such as calcified cartilage or dental tissues. Additionally, the literature is replete with reports of originally unmineralized soft-tissues preserved with ancient fossils, such as skin (Lindgren et al., 2017, 2018), feathers (Hou et al., 1995; Ji & Ji, 1996; Zhou, Barrett & Hilton, 2003) or ovarian follicles (Zheng et al., 2013; O’Connor et al., 2014). The extremely well-preserved paravians of the Jehol Biota of Northeastern China present perfect candidates to better understand differential modes of preservation, the interplay between histological and molecular preservation, and the evolution of biomolecules and tissues within the Dinosauria.

Moreover, given the current state of research on soft-tissues and biomolecules, the general questions that we ask ourselves should no longer be “*can* they preserve through deep-time?” But instead, “*how* do they preserve?” And, “*what* can these tissues and biomolecule teach us about the lives of extinct dinosaurs?” and in fact, of any other extinct organism as well.

## Additional Information and Declarations

### Data Availability

The following information was supplied regarding data availability: The data in this article are the photographs which are figures in the article.

## References

[ref-1] Adams JS, Organ CL (2005). Histologic determination of ontogenetic patterns and processes in hadrosaurian ossified tendons. Journal of Vertebrate Paleontology.

[ref-2] Agassiz L (1833–1843). Recherches sur les poissons fossiles.

[ref-3] Amprino R (1947). La structure du tissu osseux envisagée comme expression de différences dans la vitesse de l’accroissement. Archives de Biologie.

[ref-4] Anné J, Edwards NP, Wogelius RA, Tumarkin-Deratzian AR, Sellers WI, Van Veelen A, Bergmann U, Sokaras D, Alonso-Mori R, Ignatyev K (2014). Synchrotron imaging reveals bone healing and remodelling strategies in extinct and extant vertebrates. Journal of the Royal Society Interface.

[ref-5] Bailleul AM, Hall BK, Horner JR (2012). First evidence of dinosaurian secondary cartilage in the post-hatching skull of *Hypacrosaurus stebingeri* (Dinosauria, Ornithischia). PLOS ONE.

[ref-6] Bailleul AM, Hall BK, Horner JR (2013). Secondary cartilage revealed in a non-avian dinosaur embryo. PLOS ONE.

[ref-7] Bailleul AM, Holliday CM (2017). Joint histology in *Alligator mississippiensis* challenges the identification of synovial joints in fossil archosaurs and inferences of cranial kinesis. Proceedings of the Royal Society B: Biological Sciences.

[ref-8] Bailleul AM, Horner JR (2016). Comparative histology of some craniofacial sutures and skull-base synchondroses in non-avian dinosaurs and their extant phylogenetic bracket. Journal of Anatomy.

[ref-9] Bailleul AM, Nyssen-Behets C, Lengelé B, Hall BK, Horner JR (2016a). Chondroid bone in dinosaur embryos and nestlings (Ornithischia: Hadrosauridae): insights into the growth of the skull and the evolution of skeletal tissues. Comptes Rendus Palevol.

[ref-10] Bailleul AM, O’Connor J, Zhang S, Li Z, Wang Q, Lamanna MC, Zhu X, Zhou Z (2019). An early cretaceous enantiornithine (Aves) preserving an unlaid egg and probable medullary bone. Nature Communications.

[ref-11] Bailleul AM, Scannella JB, Horner JR, Evans DC (2016b). Fusion patterns in the skulls of modern archosaurs reveal that sutures are ambiguous maturity indicators for the Dinosauria. PLOS ONE.

[ref-12] Bailleul AM, Witmer LM, Holliday CM (2017). Cranial joint histology in the mallard duck (*Anas platyrhynchos*): new insights on avian cranial kinesis. Journal of Anatomy.

[ref-13] Bareggi R, Narducci P, Grill V, Sandrucci M, Bratina F (1994). On the presence of a secondary cartilage in the mental symphyseal region of human embryos and fetuses. Surgical and Radiologic Anatomy.

[ref-14] Barreto C, Albrecht RM, Bjorling DE, Horner JR, Wilsman NJ (1993). Evidence of the growth-plate and the growth of long bones in juvenile dinosaurs. Science.

[ref-15] Bertazzo S, Maidment SCR, Kallepitis C, Fearn S, Stevens MM, Xie H-N (2015). Fibres and cellular structures preserved in 75-million-year-old dinosaur specimens. Nature Communications.

[ref-265] Bloom W, Bloom MA, McLean FC (1941). Calcification and ossification. Medullary bone changes in the reproductive cycle of female pigeons. The Anatomical Record.

[ref-16] Bourdon E, Castanet J, De Ricqlès A, Scofield P, Tennyson A, Lamrous H, Cubo J (2009). Bone growth marks reveal protracted growth in New Zealand kiwi (Aves, Apterygidae). Biology Letters.

[ref-17] Bramble K, LeBlanc ARH, Lamoureux DO, Wosik M, Currie PJ (2017). Histological evidence for a dynamic dental battery in hadrosaurid dinosaurs. Scientific Reports.

[ref-18] Brink KS, Reisz RR, LeBlanc ARH, Chang RS, Lee YC, Chiang CC, Huang T, Evans DC (2015). Developmental and evolutionary novelty in the serrated teeth of theropod dinosaurs. Scientific Reports.

[ref-19] Bryant HN, Russell A, Thomason J (1995). Carnassial functioning in nimravid and felid sabertooths: theoretical basis and robustness of inferences. Functional Morphology in Vertebrate Paleontology.

[ref-20] Buckley M, Warwood S, Van Dongen B, Kitchener AC, Manning PL (2017). A fossil protein chimera; difficulties in discriminating dinosaur peptide sequences from modern cross-contamination. Proceedings of the Royal Society B: Biological Sciences.

[ref-21] Button K, You H, Kirkland JI, Zanno L (2017). Incremental growth of therizinosaurian dental tissues: implications for dietary transitions in Theropoda. PeerJ.

[ref-22] Bybee PJ, Lee AH, Lamm E-T (2006). Sizing the Jurassic theropod dinosaur *Allosaurus*: assessing growth strategy and evolution of ontogenetic scaling of limbs. Journal of Morphology.

[ref-23] Cadena EA, Schweitzer MH (2012). Variation in osteocytes morphology vs bone type in turtle shell and their exceptional preservation from the Jurassic to the present. Bone.

[ref-24] Canoville A, Schweitzer MH, Zanno LE (2019). Systemic distribution of medullary bone in the avian skeleton: ground truthing criteria for the identification of reproductive tissues in extinct Avemetatarsalia. BMC Evolutionary Biology.

[ref-25] Canoville A, Zanno L, Zheng W, Schweitzer MH (2018). New data on the skeletal distribution, microstructure, and chemistry of medullary bone in Neornithes – paleobiological implications.

[ref-26] Castanet J, Rogers KC, Cubo J, Jacques-Boisard J (2000). Periosteal bone growth rates in extant ratites (ostriche and emu). Implications for assessing growth in dinosaurs. Comptes Rendus de l’Académie des Sciences-Series III-Sciences de la Vie.

[ref-27] Cerda IA (2009). Consideraciones sobre la histogénesis de las costillas cervicales en los dinosaurios saurópodos. Ameghiniana.

[ref-28] Cerda IA, Powell JE (2010). Dermal armor histology of *Saltasaurus loricatus*, an Upper Cretaceous sauropod dinosaur from Northwest Argentina. Acta Palaeontologica Polonica.

[ref-29] Chabreck RH, Joanen T (1979). Growth rates of American alligators in Louisiana. Herpetologica.

[ref-30] Chin K, Eberth DA, Schweitzer MH, Rando TA, Sloboda WJ, Horner JR (2003). Remarkable preservation of undigested muscle tissue within a Late Cretaceous tyrannosaurid coprolite from Alberta. Canada Palaios.

[ref-31] Chinsamy A (1993). Bone histology and growth trajectory of the prosauropod dinosaur *Massospondylus carinatus*, Owen. Modern Geology.

[ref-32] Chinsamy A (2005). The microstructure of dinosaur bone: deciphering biology with fine-scale techniques.

[ref-33] Chinsamy A, Chiappe LM, Dodson P (1995). Mesozoic avian bone microstructure: physiological implications. Paleobiology.

[ref-34] Chinsamy A, Chiappe LM, Marugán-Lobón J, Chunling G, Fengjiao Z (2013). Gender identification of the Mesozoic bird *Confuciusornis sanctus*. Nature Communications.

[ref-35] Chinsamy A, Elzanowski A (2001). Bone histology: evolution of growth pattern in birds. Nature.

[ref-36] Chinsamy A, Martin LD, Dobson P (1998). Bone microstructure of the diving *Hesperornis* and the voltant *Ichthyornis* from the Niobrara Chalk of western Kansas. Cretaceous Research.

[ref-37] Coombs W (1971). The Ankylosauria.

[ref-38] Cormack DH (1987). Ham’s histology.

[ref-39] Couly GF, Coltey PM, Le Douarin NM (1993). The triple origin of skull in higher vertebrates: a study in quail-chick chimeras. Development.

[ref-40] Cubo J, Le Roy N, Martinez-Maza C, Montes L (2012). Paleohistological estimation of bone growth rate in extinct archosaurs. Paleobiology.

[ref-41] Cubo J, Woodward H, Wolff E, Horner JR (2015). First reported cases of biomechanically adaptive bone modeling in non-avian dinosaurs. PLOS ONE.

[ref-42] Dacke C, Arkle S, Cook D, Wormstone I, Jones S, Zaidi M, Bascal Z (1993). Medullary bone and avian calcium regulation. Journal of Experimental Biology.

[ref-43] Dacke CG, Elsey RM, Trosclair PL, Sugiyama T, Nevarez JG, Schweitzer MH (2015). Alligator osteoderms as a source of labile calcium for eggshell formation. Journal of Zoology.

[ref-45] De Margerie E, Cubo J, Castanet J (2002). Bone typology and growth rate: testing and quantifying ‘Amprino’s rule’ in the mallard (*Anas platyrhynchos*). Comptes Rendus Biologies.

[ref-46] De Ricqlès A (1968). Recherches paléohistologiques sur les os longs des tétrapodes: origine du tissu osseux plexiforme des dinosauriens sauropodes. Annales de Paléontologie.

[ref-47] De Ricqlès A (1969). L’histologie osseuse envisagée comme indicateur de la physiologie thermique chez les tétrapodes fossiles. Comptes Rendus Hebdomadaires des Séances de l’Academie des Sciences, Serie D: Sciences Naturelles.

[ref-48] De Ricqlès A (1972). Recherches paléohistologiques sur les os longs des Tétrapodes: Titanosuchiens, Dinocéphales et Dicynodontes. Annales de Paléontologie.

[ref-49] De Ricqlès A (1974). Recherches paléohistologiques sur les os longs des Tétrapodes: Éothériodontes et Pélycosaures. Annales de Paléontologie.

[ref-50] De Ricqlès A (1975). Recherches paléohistologiques sur les os longs des tétrapodes VII. Sur la classification, la signification fonctionnelle et l’histoire des tissus osseux des tétrapodes. Première partie, structures. Annales de Paléontologie.

[ref-51] De Ricqlès A, Bellairs AA, Cox CB (1976). On bone histology of fossil and living reptiles, with comments on its functional and evolutionary significance. Linnean Society Symposium Series 3.

[ref-52] De Ricqlès A, Thomas RDK, Olson EC (1980). Tissue structures of dinosaur bone, functional significance and possible relation to dinosaur physiology. A Cold Look at the Warm-Blooded Dinosaurs.

[ref-53] De Ricqlès A (1981). Recherches paléohistologiques sur les os longs des tétrapodes. VI. Stégocéphales. Annales de Paléontologie.

[ref-54] De Ricqlès A (2011). Vertebrate palaeohistology: past and future. Comptes Rendus Palevol.

[ref-55] De Ricqlès A, Padian K, Horner J, Gauthier J, Gall LF (2001). The bone histology of basal birds in phylogenetic and ontogenetic perspectives. New Perspectives on the Origin and Early Evolution of Birds: Proceedings of the International Symposium in Honor of John H Ostrom.

[ref-56] De Ricqlès A, Padian K, Horner JR, Francillon-Vieillot H (2000). Palaeohistology of the bones of pterosaurs (Reptilia: Archosauria): anatomy, ontogeny, and biomechanical implications. Zoological Journal of the Linnean Society.

[ref-57] De Ricqlès A, Padian K, Horner J, Lamm E-T, Myhrvold N (2003). Osteohistology of *Confuciusornis sanctus* (Theropoda: Aves). Journal of Vertebrate Paleontology.

[ref-58] De Ricqlès A, Padian K, Knoll F, Horner JR (2008). On the origin of high growth rates in archosaurs and their ancient relatives: complementary histological studies on Triassic archosauriforms and the problem of a phylogenetic signal in bone histology. Annales de Paléontologie.

[ref-59] De Ricqlès A, Suberbiola XP, Gasparini Z, Olivero E (2001). Histology of dermal ossifications in an ankylosaurian dinosaur from the Late Cretaceous of Antarctica.

[ref-60] Dubansky BH, Dubansky BD (2018). Natural development of dermal ectopic bone in the American alligator (*Alligator mississippiensis*) resembles heterotopic ossification disorders in humans. Anatomical Record.

[ref-61] Dumont M, Borbely A, Kaysser-Pyzalla A, Sander PM (2014). Long bone cortices in a growth series of *Apatosaurus* sp. (Dinosauria: Diplodocidae): geometry, body mass, and crystallite orientation of giant animals. Biological Journal of the Linnean Society.

[ref-62] Dumont M, Pyzalla A, Kostka A, Borbély A, Klein N, Remes K, Gee CT, Sander PM (2011). Characterization of sauropod bone structure. Biology of the Sauropod Dinosaurs: Understanding the Life of Giants.

[ref-63] Dumont M, Tafforeau P, Bertin T, Bhullar B-A, Field D, Schulp A, Strilisky B, Thivichon-Prince B, Viriot L, Louchart A (2016). Synchrotron imaging of dentition provides insights into the biology of *Hesperornis* and *Ichthyornis*, the last toothed birds. BMC Evolutionary Biology.

[ref-64] Dumont M, Zoeger N, Streli C, Wobrauschek P, Falkenberg G, Sander PM, Pyzalla AR (2009). Synchrotron XRF analyses of element distribution in fossilized sauropod dinosaur bones. Powder Diffraction.

[ref-65] Edmund AG (1960). Tooth replacement phenomena in the lower vertebrates. Life Science Contributions- Royal Ontario Museum.

[ref-66] Elsey RM, Wink CS (1986). The effects of estradiol on plasma calcium and femoral bone structure in alligators. Comparative Biochemistry and Physiology A.

[ref-67] Enlow DH, Brown SO (1956). A comparative histological study of fossil and recent bone tissues, Pt I. Texas Journal of Science.

[ref-68] Enlow DH, Brown SO (1957). A comparative histological study of fossil and recent bone tissues, Part II. Texas Journal of Science.

[ref-69] Enlow DH, Brown SO (1958). A comparative histological study of fossil and recent bone tissues, part III. Texas Journal of Science.

[ref-70] Enlow DH, Gans C, Bellairs A, Parsons TS (1969). The bone of reptiles. Biology of the Reptilia.

[ref-71] Erickson G (1996a). Incremental lines of von Ebner in dinosaurs and the assessment of tooth replacement rates using growth line counts. Proceedings of the National Academy of Sciences of the United States of America.

[ref-72] Erickson GM (1996b). Daily deposition of dentine in juvenile Alligator and assessment of tooth replacement rates using incremental line counts. Journal of Morphology.

[ref-73] Erickson GM, Rogers KC, Yerby SA (2001). Dinosaurian growth patterns and rapid avian growth rates. Nature.

[ref-74] Erickson GM, Krick BA, Hamilton M, Bourne GR, Norell MA, Lilleodden E, Sawyer WG (2012). Complex dental structure and wear biomechanics in hadrosaurid dinosaurs. Science.

[ref-75] Erickson GM, Makovicky PJ, Currie PJ, Norell MA, Yerby SA, Brochu CA (2004). Gigantism and comparative life-history parameters of tyrannosaurid dinosaurs. Nature.

[ref-76] Erickson GM, Rauhut OWM, Zhou Z, Turner AH, Inouye BD, Hu D, Norell MA (2009). Was dinosaurian physiology inherited by birds? Reconciling slow growth in *Archaeopteryx*. PLOS ONE.

[ref-77] Erickson GM, Rogers KC, Varricchio DJ, Norell MA, Xu X (2007). Growth patterns in brooding dinosaurs reveals the timing of sexual maturity in non-avian dinosaurs and genesis of the avian condition. Biology Letters.

[ref-78] Erickson GM, Sidebottom MA, Kay DI, Turner KT, Ip N, Norell MA, Sawyer WG, Krick BA (2015). Wear biomechanics in the slicing dentition of the giant horned dinosaur *Triceratops*. Science Advances.

[ref-79] Erickson GM, Tumanova TA (2000). Growth curve of *Psittacosaurus mongoliensis* Osborn (Ceratopsia: Psittacosauridae) inferred from long bone histology. Zoological Journal of the Linnean Society.

[ref-80] Erickson GM, Zelenitsky DK, Kay DI, Norell MA (2017). Dinosaur incubation periods directly determined from growth-line counts in embryonic teeth show reptilian-grade development. Proceedings of the National Academy of Sciences of the United States of America.

[ref-81] Falcon-Lang HJ, Digrius DM (2014). Palaeobotany under the microscope: history of the invention and widespread adoption of the petrographic thin section technique. Quekett Journal of Microscopy.

[ref-82] Farke AA, Chok DJ, Herrero A, Scolieri B, Werning S (2013). Ontogeny in the tube-crested dinosaur *Parasaurolophus* (Hadrosauridae) and heterochrony in hadrosaurids. PeerJ.

[ref-83] Francillon-Vieillot H, De Buffrénil V, Castanet J, Géraudie J, Meunier F, Sire J, Zylberberg L, De Ricqlès A, Carter JG (1990). Microstructure and mineralization of vertebrate skeletal tissues. Skeletal Biomineralization, Patterns, Processes and Evolutionary Trends.

[ref-84] Gaengler P, Teaford MF, Smith MM, Ferguson MWJ (2000). Evolution of tooth attachment in lower vertebrates to tetrapods. Development, Function and Evolution of Teeth.

[ref-85] Gao C, Chiappe LM, Zhang F, Pomeroy DL, Shen C, Chinsamy A, Walsh MO (2012). A subadult specimen of the Early Cretaceous bird *Sapeornis chaoyangensis* and a taxonomic reassessment of sapeornithids. Journal of Vertebrate Paleontology.

[ref-86] García RA, Zurriaguz V (2016). Histology of teeth and tooth attachment in titanosaurs (Dinosauria; Sauropoda). Cretaceous Research.

[ref-87] Garilli V, Klein N, Buffetaut E, Sander PM, Pollina F, Galletti L, Cillari A, Guzzetta D (2009). First dinosaur bone from Sicily identified by histology and its paleobiogeographical implications. Neues Jahrbuch für Geologie und Paläontologie - Abhandlungen.

[ref-88] Garland AN (1989). Microscopical analysis of fossil bone. Applied Geochemistry.

[ref-89] Goodwin MB, Evans DC (2016). The early expression of squamosal horns and parietal ornamentation confirmed by new end-stage juvenile *Pachycephalosaurus* fossils from the Upper Cretaceous Hell Creek Formation, Montana. Journal of Vertebrate Paleontology.

[ref-90] Goodwin MB, Horner JR (2004). Cranial histology of pachycephalosaurs (Ornithischia: Marginocephalia) reveals transitory structures inconsistent with head-butting behavior. Paleobiology.

[ref-91] Grady JM, Enquist BJ, Dettweiler-Robinson E, Wright NA, Smith FA (2014). Evidence for mesothermy in dinosaurs. Science.

[ref-92] Griebeler EM, Klein N, Sander PM (2013). Aging, maturation and growth of sauropodomorph dinosaurs as deduced from growth curves using long bone histological data. PLOS ONE.

[ref-93] Grigg G, Kirshner D (2015). Biology and evolution of crocodylians.

[ref-94] Gross W (1934). Die Typen des mikroskopischen Knochenbaues bei fossilen Stegocephalen und Reptilien. Zeitschrift für Anatomie und Entwicklungsgeschichte.

[ref-95] Hagelberg E, Bell LS, Allen T, Boyde A, Jones SJ, Clegg JB (1991). Analysis of ancient bone DNA: techniques and applications. Philosophical Transactions of the Royal Society of London B: Biological Sciences.

[ref-96] Haines RW, Mohuiddin A (1968). Metaplastic bone. Journal of Anatomy.

[ref-97] Hall BK (1967). The distribution and fate of the adventitious cartilage in the skull of the eastern rosella, *Platycerus eximius* (Aves: Psittaciformes). Australian Journal of Zoology.

[ref-98] Hall BK (1968). The fate of adventitious and embryonic articular cartilage in the skull of the common fowl, *Gallus domesticus* (Aves: Phasianidae). Australian Journal of Zoology.

[ref-99] Hall BK (1972). Immobilization and cartilage transformation into bone in the embryonic chick. Anatomical Record.

[ref-100] Hall BK (2000). The evolution of the neural crest in vertebrates. Regulatory Processes in Development.

[ref-267] Hall BK (2005). Bones and cartilage: developmental and evolutionary skeletal biology.

[ref-101] Hayashi S, Carpenter K, Scheyer TM, Watabe M, Suzuki D (2010). Function and evolution of ankylosaur dermal armor. Acta Palaeontologica Polonica.

[ref-102] Hayashi S, Carpenter K, Watabe M, McWhinney LA (2012). Ontogenetic histology of *Stegosaurus* plates and spikes. Palaeontology.

[ref-103] Haynes S, Searle JB, Bretman A, Dobney KM (2002). Bone preservation and ancient DNA: the application of screening methods for predicting DNA survival. Journal of Archaeological Science.

[ref-104] Heck CT, Varricchio DJ, Gaudin TJ, Woodward HN, Horner JR (2019). Ontogenetic changes in the long bone microstructure in the nine-banded armadillo (*Dasypus novemcinctus*). PLOS ONE.

[ref-105] Hedges REM (2002). Bone diagenesis: an overview of processes. Archaeometry.

[ref-269] Hedges SB, Schweitzer MH, Henikoff S, Allard MW, Young D, Huyen Y, Zischler H, Höss M, Handt O, von Haeseler A (1995). Detecting dinosaur DNA. Science.

[ref-106] Hems T, Tillmann B (2000). Tendon entheses of the human masticatory muscles. Anatomy and Embryology.

[ref-107] Hieronymus TL (2006). Quantitative microanatomy of jaw muscle attachment in extant diapsids. Journal of Morphology.

[ref-108] Hieronymus TL, Witmer LM, Tanke DH, Currie PJ (2009). The facial integument of centrosaurine ceratopsids: morphological and histological correlates of novel skin structures. Anatomical Record.

[ref-109] Hofmann R, Stein K, Sander PM (2014). Constraints on the lamina density of laminar bone architecture of large-bodied dinosaurs and mammals. Acta Palaeontologica Polonica.

[ref-111] Horner JR, Currie PJ, Carpenter K, Hirsch KF, Horner JR (1994). Embryonic and neonatal morphology and ontogeny of a new species of *Hypacrosaurus* (Ornithischia, Lambeosauridae) from Montana and Alberta. Dinosaur Eggs and Babies.

[ref-112] Horner JR, De Ricqlès AJ, Padian K (1999). Variation in skeletochronological indicators of the hadrosaurid dinosaur *Hypacrosaurus*: implications for age assessment of dinosaurs. Paleobiology.

[ref-113] Horner JR, De Ricqlès A, Padian K (2000). Long bone histology of the hadrosaurid dinosaur *Maiasaura peeblesorum*: growth dynamics and physiology based on an ontogenetic series of skeletal elements. Journal of Vertebrate Paleontology.

[ref-114] Horner JR, De Ricqlès A, Padian K, Scheetz RD (2009). Comparative long bone histology and growth of the hypsilophodontid dinosaurs *Orodromeus makelai*, *Dryosaurus altus*, and *Tenontosaurus tillettii* (Ornithischia: Euornithopoda). Journal of Vertebrate Paleontology.

[ref-115] Horner JR, Goodwin MB (2009). Extreme cranial ontogeny in the Upper Cretaceous dinosaur *Pachycephalosaurus*. PLOS ONE.

[ref-116] Horner JR, Lamm E-T (2011). Ontogeny of the parietal frill of *Triceratops*: a preliminary histological analysis. Comptes Rendus Palevol.

[ref-117] Horner JR, Padian K (2004). Age and growth dynamics of *Tyrannosaurus rex*. Proceedings of the Royal Society B: Biological Sciences.

[ref-118] Horner JR, Padian K, De Ricqlès A (2001). Comparative osteohistology of some embryonic and perinatal archosaurs: developmental and behavioral implications for dinosaurs. Paleobiology.

[ref-119] Horner JR, Woodward HN, Bailleul AM (2016). Mineralized tissues in dinosaurs interpreted as having formed through metaplasia: a preliminary evaluation. Comptes Rendus Palevol.

[ref-120] Hou L-H, Zhou Z, Martin LD, Feduccia A (1995). A beaked bird from the Jurassic of China. Nature.

[ref-121] Hübner TR (2012). Bone histology in *Dysalotosaurus lettowvorbecki* (Ornithischia: Iguanodontia)—variation, growth, and implications. PLOS ONE.

[ref-122] Huttenlocker AK, Woodward HN, Hall BK, Padian K, Lamm E-T (2013). The biology of bone. Bone Histology of Fossil Tetrapods.

[ref-123] Hwang SH (2005). Phylogenetic patterns of enamel microstructure in dinosaur teeth. Journal of Morphology.

[ref-124] Hwang SH (2011). The evolution of dinosaur tooth enamel microstructure. Biological Reviews.

[ref-125] Ji Q, Ji S (1996). On the discovery of the earliest fossil bird in China (*Sinosauropteryx* gen. nov.) and the origin of birds. Chinese Geology.

[ref-126] Jiang B, Zhao T, Regnault S, Edwards NP, Kohn SC, Li Z, Wogelius RA, Benton MJ, Hutchinson JR (2017). Cellular preservation of musculoskeletal specializations in the Cretaceous bird *Confuciusornis*. Nature Communications.

[ref-127] Klein N, Christian A, Sander PM (2012). Histology shows that elongated neck ribs in sauropod dinosaurs are ossified tendons. Biology Letters.

[ref-128] Klein N, Sander PM (2007). Bone histology and growth of the prosauropod *Plateosaurus engelhardti* Meyer, 1837 from the Norian bonebeds of Trossingen (Germany) and Frick (Switzerland). Special Papers in Palaeontology.

[ref-129] Klein N, Sander M (2008). Ontogenetic stages in the long bone histology of sauropod dinosaurs. Paleobiology.

[ref-130] Klein N, Sander PM, Stein K, Loeuff JL, Carballido JL, Eric B (2012). Modified laminar bone in *Ampelosaurus atacis* and other titanosaurs (Sauropoda): implications for life history and physiology. PLOS ONE.

[ref-131] Knoll F, Chiappe LM, Sanchez S, Garwood RJ, Edwards NP, Wogelius RA, Sellers WI, Manning PL, Ortega F, Serrano FJ (2018). A diminutive perinate European Enantiornithes reveals an asynchronous ossification pattern in early birds. Nature Communications.

[ref-132] Koenigswald WV, Sander PM, Koenigswald WV, Sander PM (1997). Glossary of terms used for enamel microstructure. Tooth Enamel Microstructure.

[ref-133] Köhler M, Marín-Moratalla N, Jordana X, Aanes R (2012). Seasonal bone growth and physiology in endotherms shed light on dinosaur physiology. Nature.

[ref-134] Kvam T (1960). The teeth of *Alligator mississippiensis* Daud: VI. Periodontium. Acta Odontologica Scandinavica.

[ref-135] Lambertz M, Bertozzo F, Sander PM (2018). Bone histological correlates for air sacs and their implications for understanding the origin of the dinosaurian respiratory system. Biology Letters.

[ref-136] Lamm E-T, Padian K, Lamm E-T (2013). Preparation and sectioning of specimens. Bone Histology of Fossil Tetrapods: Advancing Methods, Analysis, and Interpretation.

[ref-137] Landis WJ, Silver FH (2002). The structure and function of normally mineralizing avian tendons. Comparative Biochemistry and Physiology Part A: Molecular & Integrative Physiology.

[ref-138] LeBlanc ARH, Reisz RR, Evans DC, Bailleul AM (2016). Ontogeny reveals function and evolution of the hadrosaurid dinosaur dental battery. BMC Evolutionary Biology.

[ref-139] Lee AH, Werning S (2008). Sexual maturity in growing dinosaurs does not fit reptilian growth models. Proceedings of the National Academy of Sciences of the United States of America.

[ref-140] Legendre LJ, Guénard G, Botha-Brink J, Cubo J (2016). Palaeohistological evidence for ancestral high metabolic rate in archosaurs. Systematic Biology.

[ref-141] Lehman TM, Woodward HN (2008). Modeling growth rates for sauropod dinosaurs. Paleobiology.

[ref-142] Lessner EJ, Gant CA, Hieronymus TL, Vickaryous MK, Holliday CM (2019). Anatomy and ontogeny of the mandibular symphysis in *Alligator mississippiensis*. Anatomical Record.

[ref-143] Lindahl T (1993). Instability and decay of the primary structure of DNA. Nature.

[ref-144] Lindgren J, Kuriyama T, Madsen H, Sjövall P, Zheng W, Uvdal P, Engdahl A, Moyer AE, Gren JA, Kamezaki N (2017). Biochemistry and adaptive colouration of an exceptionally preserved juvenile fossil sea turtle. Scientific Reports.

[ref-145] Lindgren J, Moyer A, Schweitzer MH, Sjövall P, Uvdal P, Nilsson DE, Heimdal J, Engdahl A, Gren JA, Schultz BP (2015). Interpreting melanin-based coloration through deep time: a critical review. Proceedings of the Royal Society B: Biological Sciences.

[ref-146] Lindgren J, Sjövall P, Thiel V, Zheng W, Ito S, Wakamatsu K, Hauff R, Kear BP, Engdahl A, Alwmark C, Eriksson ME, Jarenmark M, Sachs S, Ahlberg PE, Marone F, Kuriyama T, Gustafsson O, Malmberg P, Thomen A, Rodríguez-Meizoso I, Uvdal P, Ojika M, Schweitzer MH (2018). Soft-tissue evidence for homeothermy and crypsis in a Jurassic ichthyosaur. Nature.

[ref-147] Lindgren J, Uvdal P, Engdahl A, Lee AH, Alwmark C, Bergquist K-E, Nilsson E, Ekström P, Rasmussen M, Douglas DA (2011). Microspectroscopic evidence of Cretaceous bone proteins. PLOS ONE.

[ref-148] Main RP, De Ricqlès A, Horner JR, Padian K (2005). The evolution and function of thyreophoran dinosaur scutes: implications for plate function in stegosaurs. Paleobiology.

[ref-149] Mantell GA (1850a). On a dorsal dermal spine of the *Hylæosaurus*, recently discovered in the strata of Tilgate Forest. Philosophical Transactions of the Royal Society of London.

[ref-150] Mantell GA (1850b). On the *Pelorosaurus*; an undescribed gigantic terrestrial reptile whose remains are associated with those of the iguanodon and other saurians in the strata of Tilgate Forest, Sussex. Philosophical Transactions of the Royal Society of London.

[ref-151] McIntosh JE, Anderton X, Flores-De-Jacoby L, Carlson DS, Shuler CF, Diekwisch TG (2002). Caiman periodontium as an intermediate between basal vertebrate ankylosis-type attachment and mammalian true periodontium. Microscopy Research and Technique.

[ref-152] McNamara ME, Orr PJ, Kearns SL, Alcalá L, Anadón P, Penalver Molla E (2009). Soft-tissue preservation in Miocene frogs from Libros, Spain: insights into the genesis of decay microenvironments. Palaios.

[ref-153] McNamara M, Orr PJ, Kearns SL, Alcalá L, Anadón P, Peñalver-Mollá E (2010). Organic preservation of fossil musculature with ultracellular detail. Proceedings of the Royal Society B: Biological Sciences.

[ref-154] McNamara ME, Zhang F, Kearns SL, Orr PJ, Toulouse A, Foley T, Hone DWE, Rogers CS, Benton MJ, Johnson D, Xu X, Zhou Z (2018). Fossilized skin reveals coevolution with feathers and metabolism in feathered dinosaurs and early birds. Nature Communications.

[ref-155] Mescher A (2013). Junqueira’s basic histology: text and atlas.

[ref-156] Mitchell J, Legendre LJ, Lefèvre C, Cubo J (2017). Bone histological correlates of soaring and high-frequency flapping flight in the furculae of birds. Zoology.

[ref-157] Mitchell J, Sander PM (2014). The three-front model: a developmental explanation of long bone diaphyseal histology of Sauropoda. Biological Journal of the Linnean Society.

[ref-158] Mitchell J, Sander PM, Stein K (2017). Can secondary osteons be used as ontogenetic indicators in sauropods? Extending the histological ontogenetic stages into senescence. Paleobiology.

[ref-160] Montes L, Le Roy N, Perret M, De Buffrenil V, Castanet J, Cubo J (2007). Relationships between bone growth rate, body mass and resting metabolic rate in growing amniotes: a phylogenetic approach. Biological Journal of the Linnean Society.

[ref-161] Moyer AE, Zheng W, Schweitzer MH (2016). Keratin durability has implications for the fossil record: results from a 10 year feather degradation experiment. PLOS ONE.

[ref-162] Murray PDF (1963). Adventitious (secondary) cartilage in the chick embryo: and the development of certain bones and articulations in the chick skull. Australian Journal of Zoology.

[ref-163] Murray PDF, Drachman DB (1969). The role of movement in the development of joints and related structures: the head and neck in the chick embryo. Development.

[ref-164] Musumeci G (2014). Past, present and future: overview on histology and histopathology. Journal of Histology and Histopathology.

[ref-165] Myhrvold NP (2016). Dinosaur metabolism and the allometry of maximum growth rate. PLOS ONE.

[ref-166] Nopcsa FB (1933). On the histology of-the ribs in immature and half-grown trachodont dinosaurs. Proceedings of the Zoological Society of London.

[ref-167] O’Connor J, Erickson GM, Norell M, Bailleul AM, Hu H, Zhou Z (2018). Medullary bone in an Early Cretaceous enantiornithine (Aves) and discussion regarding its identification in fossils. Nature Communications.

[ref-168] O’Connor JK, Wang M, Zheng X-T, Wang X-L, Zhou Z-H (2014). The histology of two female Early Cretaceous birds. Vertebrata Palasiatica.

[ref-169] O’Connor JK, Wang M, Zhou S, Zhou Z (2015). Osteohistology of the Lower Cretaceous Yixian Formation ornithuromorph (Aves) *Iteravis huchzermeyeri*. Palaeontologia Electronica.

[ref-170] O’Connor JK, Zheng X, Wang X, Wang Y, Zhou Z (2013). Ovarian follicles shed new light on dinosaur reproduction during the transition towards birds. National Science Review.

[ref-171] Orlando L, Ginolhac A, Raghavan M, Vilstrup J, Rasmussen M, Magnussen K, Steinmann KE, Kapranov P, Thompson JF, Zazula G, Froese D, Moltke I, Shapiro B, Hofreiter M, Al-Rasheid KAS, Gilbert MTP, Willerslev E (2011). True single-molecule DNA sequencing of a pleistocene horse bone. Genome Research.

[ref-172] Ørvig T (1951). Histologic studies of Placoderms and fossil Elasmobranchs. The endoskeleton, with remarks on the hard tissues of lower vertebrates in general. Arkiv för zoologi.

[ref-173] Padian K, De Ricqlès AJ, Horner JR (2001). Dinosaurian growth rates and bird origins. Nature.

[ref-174] Padian K, Horner JR (2011). The evolution of ‘bizarre structures’ in dinosaurs: biomechanics, sexual selection, social selection or species recognition?. Journal of Zoology.

[ref-175] Padian K, Horner JR, De Ricqlès A (2004). Growth in small dinosaurs and pterosaurs: the evolution of archosaurian growth strategies. Journal of Vertebrate Paleontology.

[ref-176] Padian K, Lamm ET (2013). Bone histology of fossil tetrapods: advancing methods, analysis, and interpretation.

[ref-177] Pan Y, Hu L, Zhao T (2018). Applications of chemical imaging techniques in paleontology. National Science Reviews.

[ref-178] Pan Y, Zheng W, Moyer AE, O’Connor JK, Wang M, Zheng X, Wang X, Schroeter ER, Zhou Z, Schweitzer MH (2016). Molecular evidence of keratin and melanosomes in feathers of the Early Cretaceous bird Eoconfuciusornis. Proceedings of the National Academy of Sciences of the United States of America.

[ref-179] Pawlicki R (1977). Topochemical localization of lipids in dinosaur bone by means of Sudan B black. Acta Histochemica.

[ref-180] Pawlicki R, Korbel A, Kubiak H (1966). Cells, collagen fibrils and vessels in dinosaur bone. Nature.

[ref-181] Pawlicki R, Nowogrodzka-Zagórska M (1998). Blood vessels and red blood cells preserved in dinosaur bones. Annals of Anatomy.

[ref-182] Persson M (1983). The role of movements in the development of sutural and diarthrodial joints tested by long-term paralysis of chick embryos. Journal of Anatomy.

[ref-183] Petermann H, Sander M (2013). Histological evidence for muscle insertion in extant amniote femora: implications for muscle reconstruction in fossils. Journal of Anatomy.

[ref-184] Ponton F, Elżanowski A, Castanet J, Chinsamy A, De Margerie E, De Ricqlès A, Cubo J (2004). Variation of the outer circumferential layer in the limb bones of birds. Acta Ornithologica.

[ref-185] Ponton F, Montes L, Castanet J, Cubo J (2007). Bone histological correlates of high-frequency flapping flight and body mass in the furculae of birds: a phylogenetic approach. Biological Journal of the Linnean Society.

[ref-186] Prondvai E (2017). Medullary bone in fossils: function, evolution and significance in growth curve reconstructions of extinct vertebrates. Journal of Evolutionary Biology.

[ref-187] Prondvai E, Godefroit P, Adriaens D, Hu D-Y (2018). Intraskeletal histovariability, allometric growth patterns, and their functional implications in bird-like dinosaurs. Scientific Reports.

[ref-188] Prondvai E, Stein KHW (2014). Medullary bone-like tissue in the mandibular symphyses of a pterosaur suggests non-reproductive significance. Scientific Reports.

[ref-189] Prondvai E, Stein KH, De Ricqlès A, Cubo J (2014). Development-based revision of bone tissue classification: the importance of semantics for science. Biological Journal of the Linnean Society.

[ref-190] Quekett J (1849). On the intimate structure of bone, as composing the skeleton, in the four great classes of animals, viz., mammals, birds, reptiles, and fishes, with some remarks on the great value of the knowledge of such structure in determining the affinities of minute fragments of organic remains. Transactions of the Microscopical Society of London.

[ref-191] Redelstorff R, Hübner TR, Chinsamy A, Sander PM (2013). Bone histology of the stegosaur *Kentrosaurus aethiopicus* (Ornithischia: Thyreophora) from the Upper Jurassic of Tanzania. Anatomical Record.

[ref-192] Redelstorff R, Sander PM (2009). Long and girdle bone histology of *Stegosaurus*: implications for growth and life history. Journal of Vertebrate Paleontology.

[ref-193] Reid R (1996). Bone histology of the Cleveland-Lloyd dinosaurs and of dinosaurs in general, Part I: Introduction: Introduction to bone tissues. Brigham Young University Geology Studies.

[ref-194] Reisz RR, Huang TD, Roberts EM, Peng SR, Sullivan C, Stein K, LeBlanc ARH, Shieh DB, Chang RS, Chiang CC, Yang C, Zhong S (2013). Embryology of Early Jurassic dinosaur from China with evidence of preserved organic remains. Nature.

[ref-195] Ricklefs RE (1968). Patterns of growth in birds. Ibis.

[ref-196] Rogers KC, D’Emic M, Rogers R, Vickaryous M, Cagan A (2011). Sauropod dinosaur osteoderms from the Late Cretaceous of Madagascar. Nature Communications.

[ref-197] Rogers KC, Erickson GM, Rogers KC, Wilson JA (2005). Sauropod histology. The Sauropods, Evolution and Paleobiology.

[ref-198] Rogers KC, Whitney M, D’Emic M, Bagley B (2016). Precocity in a tiny titanosaur from the Cretaceous of Madagascar. Science.

[ref-199] Sanchez S, Ahlberg PE, Trinajstic KM, Mirone A, Tafforeau P (2012). Three-dimensional synchrotron virtual paleohistology: a new insight into the world of fossil bone microstructures. Microscopy and Microanalysis.

[ref-200] Sander PM (1999). The microstructure of reptilian tooth enamel: terminology, function, and phylogeny. Münchner geowissenschaftliche Abhandlungen.

[ref-201] Sander PM (2000a). Longbone histology of the Tendaguru sauropods: implications for growth and biology. Paleobiology.

[ref-202] Sander P, Teaford M, Ferguson MWJ, Smith MM (2000b). Prismless enamel in amniotes: terminology, function, and evolution. Development, Function and Evolution of Teeth.

[ref-203] Sander PM, Andrassy P (2006). Lines of arrested growth and long bone histology in Pleistocene large mammals from Germany: what do they tell us about dinosaur physiology?. Palaeontographica A.

[ref-204] Sander PM, Klein N (2005). Developmental plasticity in the life history of a prosauropod dinosaur. Science.

[ref-205] Sander PM, Klein N, Buffetaut E, Cuny G, Suteethorn V, Loeuff JLe (2004). Adaptive radiation in sauropod dinosaurs: bone histology indicates rapid evolution of giant body size through acceleration. Organisms Diversity & Evolution.

[ref-206] Sander PM, Klein N, Stein K, Wings O, Klein N, Remes K, Gee CT, Sander PM (2011). Sauropod bone histology and implications for sauropod biology. Biology of the Sauropod Dinosaurs. Understanding the Life of Giants.

[ref-207] Sander PM, Mateus O, Laven T, Knötschke N (2006). Bone histology indicates insular dwarfism in a new Late Jurassic sauropod dinosaur. Nature.

[ref-208] Sato T, Cheng Y, Wu X, Zelenitsky DK, Hsiao Y (2005). A pair of shelled eggs inside a female dinosaur. Science.

[ref-209] Scannella JB, Horner JR (2010). *Torosaurus* Marsh, 1891, is *Triceratops* Marsh, 1889 (Ceratopsidae: Chasmosaurinae): synonymy through ontogeny. Journal of Vertebrate Paleontology.

[ref-210] Scannella JB, Horner JR (2011). *Nedoceratops*’: an example of a transitional morphology. PLOS ONE.

[ref-211] Scheyer TM, Desojo JB, Cerda IA (2014). Bone histology of phytosaur, aetosaur, and other archosauriform osteoderms (Eureptilia, Archosauromorpha). Anatomical Record.

[ref-268] Scheyer TM, Martin Sander P, Joyce WG, Böhme W, Witzel U (2007). A plywood structure in the shell of fossil and living soft-shelled turtles (Trionychidae) and its evolutionary implications. Organisms Diversity & Evolution.

[ref-212] Scheyer TM, Sander PM (2004). Histology of ankylosaur osteoderms: implications for systematics and function. Journal of Vertebrate Paleontology.

[ref-213] Schweitzer MH, Avci R, Collier T, Goodwin MB (2008). Microscopic, chemical and molecular methods for examining fossil preservation. Comptes Rendus Palevol.

[ref-214] Schweitzer M, Chiappe L, Garrido A, Lowenstein J, Pincus S (2005b). Molecular preservation in Late Cretaceous sauropod dinosaur eggshells. Proceedings of the Royal Society B: Biological Sciences.

[ref-215] Schweitzer MH, Elsey RM, Dacke CG, Horner JR, Lamm E-T (2007). Do egg-laying crocodilian (*Alligator mississippiensis*) archosaurs form medullary bone?. Bone.

[ref-216] Schweitzer MH, Horner JR (1999). Intravascular microstructures in trabecular bone tissues of *Tyrannosaurus rex*. Annales de Paléontologie.

[ref-217] Schweitzer MH, Johnson C, Zocco TG, Horner JR, Starkey JR (1997a). Preservation of biomolecules in cancellous bone of *Tyrannosaurus rex*. Journal of Vertebrate Paleontology.

[ref-218] Schweitzer MH, Marshall M, Carron K, Bohle DS, Busse SC, Arnold EV, Barnard D, Horner JR, Starkey JR (1997b). Heme compounds in dinosaur trabecular bone. Proceedings of the National Academy of Sciences of the United States of America.

[ref-219] Schweitzer MH, Watt JA, Avci R, Forster CA, Krause DW, Knapp L, Rogers RR, Beech I, Marshall M (1999b). Keratin immunoreactivity in the Late Cretaceous bird *Rahonavis ostromi*. Journal of Vertebrate Paleontology.

[ref-220] Schweitzer MH, Watt J, Avci R, Knapp L, Chiappe L, Norell M, Marshall M (1999a). Beta-keratin specific immunological reactivity in feather-like structures of the Cretaceous Alvarezsaurid, *Shuvuuia deserti*. Journal of Experimental Zoology.

[ref-221] Schweitzer MH, Wittmeyer JL, Horner JR (2005). Gender-specific reproductive tissue in ratites and *Tyrannosaurus rex*. Science.

[ref-222] Schweitzer MH, Wittmeyer JL, Horner JR (2007). Soft tissue and cellular preservation in vertebrate skeletal elements from the Cretaceous to the present. Proceedings of the Royal Society B: Biological Sciences.

[ref-223] Schweitzer MH, Wittmeyer JL, Horner JR, Toporski JK (2005a). Soft-tissue vessels and cellular preservation in *Tyrannosaurus rex*. Science.

[ref-224] Schweitzer MH, Zheng W, Cleland TP, Bern M (2013). Molecular analyses of dinosaur osteocytes support the presence of endogenous molecules. Bone.

[ref-270] Schweitzer MH, Zheng W, Cleland TP, Goodwin MB, Boatman E, Theil E, Marcus MA, Fakra SC (2014). A role for iron and oxygen chemistry in preserving soft tissues, cells and molecules from deep time. Proceedings of the Royal Society B: Biological Sciences.

[ref-225] Schweitzer MH, Zheng W, Zanno L, Werning S, Sugiyama T (2016). Chemistry supports the identification of gender-specific reproductive tissue in *Tyrannosaurus rex*. Scientific Reports.

[ref-226] Seitz ALL (1907). Vergleichende Studien über den mikroskopischen Knochenbau fossiler und rezenter Reptilien, und dessen Bedeutung für das Wachstum und Umbildung des Knochengewebes im allgemeinen. Abhandlungen der kaiserlichen Leopold Carolingischen deutschen Akademie der Naturforscher, Nova Acta.

[ref-227] Stein K, Csiki Z, Rogers KC, Weishampel DB, Redelstorff R, Carballido JL, Sander PM (2010). Small body size and extreme cortical bone remodeling indicate phyletic dwarfism in *Magyarosaurus dacus* (Sauropoda: Titanosauria). Proceedings of the National Academy of Sciences of the United States of America.

[ref-228] Stein M, Hayashi S, Sander PM (2013). Long bone histology and growth patterns in ankylosaurs: implications for life history and evolution. PLOS ONE.

[ref-229] Stein K, Sander PM (2009). Histological core drilling: a less destructive method for studying bone histology.

[ref-230] Stout SD (1978). Histological structure and its preservation in ancient bone. Current Anthropology.

[ref-231] Tadokoro O, Mishima H, Maeda T, Kozawa Y (1998). Innervation of the periodontal ligament in the alligatorid *Caiman crocodilus*. European Journal of Oral Sciences.

[ref-266] Taylor T, Moore J (1953). Avian medullary bone. Nature.

[ref-232] Tsai HP, Holliday CM (2015). Articular soft tissue anatomy of the archosaur hip joint: structural homology and functional implications. Journal of Morphology.

[ref-233] Tumarkin-Deratzian AR, Ryan M, Chinnery-Allgeier B, Eberth D (2010). Histological evaluation of ontogenetic bone surface texture changes in the frill of *Centrosaurus apertus*.

[ref-234] Vanderven E, Burns ME, Currie PJ (2014). Histologic growth dynamic study of *Edmontosaurus regalis* (Dinosauria: Hadrosauridae) from a bonebed assemblage of the Upper Cretaceous Horseshoe Canyon Formation. Canadian Journal of Earth Sciences.

[ref-235] Varricchio DJ (1993). Bone microstructure of the Upper Cretaceous theropod dinosaur *Troodon formosus*. Journal of Vertebrate Paleontology.

[ref-236] Vickaryous MK, Hall BK (2008). Development of the dermal skeleton in *Alligator mississippiensis* (Archosauria, Crocodylia) with comments on the homology of osteoderms. Journal of Morphology.

[ref-237] Vickaryous M, Russell A, Currie PJ, Carpenter K (2001). Cranial ornamentation of ankylosaurs (Ornithischia: Thyreophora) reappraisal of developmental hypotheses. The Armored Dinosaurs.

[ref-238] Vinther J, Briggs DE, Prum RO, Saranathan V (2008). The colour of fossil feathers. Biology Letters.

[ref-239] Wang J, Hao X, Kundrát M, Liu Z, Uesugi K, Jurašeková Z, Guo B, Hoshino M, Li Y, Monfroy Q, Zhou B, Fabriciová G, Kang A, Wang M, Si Y, Gao J, Xu G, Li Z (2019). Bone tissue histology of the Early Cretaceous bird *Yanornis*: evidence for a diphyletic origin of modern avian growth strategies within Ornithuromorpha. Historical Biology.

[ref-240] Wang X, O’Connor JK, Maina JN, Pan Y, Wang M, Wang Y, Zheng X, Zhou Z (2018). *Archaeorhynchus* preserving significant soft tissue including probable fossilized lungs. Proceedings of the National Academy of Sciences of the United States of America.

[ref-241] Wang M, Zhou Z (2017). A new adult specimen of the basalmost ornithuromorph bird *Archaeorhynchus spathula* (Aves: Ornithuromorpha) and its implications for early avian ontogeny. Journal of Systematic Palaeontology.

[ref-242] Waskow K, Mateus O (2017). Dorsal rib histology of dinosaurs and a crocodylomorph from western Portugal: skeletochronological implications on age determination and life history traits. Comptes Rendus Palevol.

[ref-243] Waskow K, Sander PM (2014). Growth record and histological variation in the dorsal ribs of *Camarasaurus* sp.(Sauropoda). Journal of Vertebrate Paleontology.

[ref-244] Werner J, Griebeler EM (2014). Allometries of maximum growth rate versus body mass at maximum growth indicate that non-avian dinosaurs had growth rates typical of fast growing ectothermic sauropsids. PLOS ONE.

[ref-245] Werning S (2018). Medullary bone is phylogenetically widespread and its skeletal distribution varies by taxon. Journal of Ornithology.

[ref-246] Westergaard B, Ferguson MW (1990). Development of the dentition in *Alligator mississippiensis*: upper jaw dental and craniofacial development in embryos, hatchlings, and young juveniles, with a comparison to lower jaw development. American Journal of Anatomy.

[ref-247] Wiemann J, Fabbri M, Yang T-R, Stein K, Sander PM, Norell MA, Briggs DEG (2018). Fossilization transforms vertebrate hard tissue proteins into N-heterocyclic polymers. Nature Communications.

[ref-248] Wiemann J, Yang T-R, Norell MA (2018). Dinosaur egg colour had a single evolutionary origin. Nature.

[ref-249] Willerslev E, Cooper A (2005). Ancient DNA. Proceedings of the Royal Society B: Biological Sciences.

[ref-250] Witmer LM, Thomason J (1995). The Extant Phylogenetic Bracket and the importance of reconstructing soft tissues in fossils. Functional Morphology in Vertebrate Paleontology.

[ref-251] Wogelius RA, Manning PL, Barden HE, Edwards NP, Webb SM, Sellers WI, Taylor KG, Larson PL, Dodson P, You H, Da-qing L, Bergmann U (2011). Trace metals as biomarkers for eumelanin pigment in the fossil record. Science.

[ref-252] Wolf D, Kalthoff DC, Sander PM (2012). Osteoderm histology of the Pampatheriidae (Cingulata, Xenarthra, Mammalia): implications for systematics, osteoderm growth, and biomechanical adaptation. Journal of Morphology.

[ref-253] Woodward HN (2019). *Maiasaura* (Dinosauria: Hadrosauridae) tibia osteohistology reveals non-annual cortical vascular rings in young of the year. Frontiers in Earth Sciences.

[ref-254] Woodward HN, Fowler EAF, Farlow JO, Horner JR (2015). *Maiasaura*, a model organism for extinct vertebrate population biology: a large sample statistical assessment of growth dynamics and survivorship. Paleobiology.

[ref-255] Woodward HN, Horner JR, Farlow JO (2011). Osteohistological evidence for determinate growth in the American alligator. Journal of Herpetology.

[ref-256] Woodward H, Padian K, Lee A, Padian K, Lamm E-T (2013). Skeletochronology. Bone Histology of Fossil Tetrapods: Advancing Methods, Analysis, and Interpretation.

[ref-257] Yamamoto T, Nakamura H, Tsuji T, Hirata A (2001). Ultracytochemical study of medullary bone calcification in estrogen injected male Japanese quail. Anatomical Record.

[ref-258] Zhang F, Hou L, Ouyang L (1998). Osteological microstructure of *Confuciusornis*: preliminary report. Vertebrata Palasiatica.

[ref-259] Zhao Q, Benton MJ, Sullivan C, Sander PM, Xu X (2013). Histology and postural change during the growth of the ceratopsian dinosaur *Psittacosaurus lujiatunensis*. Nature Communications.

[ref-260] Zheng X, O’Connor J, Huchzermeyer F, Wang X, Wang Y, Wang M, Zhou Z (2013). Preservation of ovarian follicles reveals early evolution of avian reproductive behaviour. Nature.

[ref-261] Zheng X, O’Connor JK, Wang X, Pan Y, Wang Y, Wang M, Zhou Z (2017). Exceptional preservation of soft tissue in a new specimen of *Eoconfuciusornis* and its biological implications. National Science Review.

[ref-264] Zheng X, O’Connor J, Wang X, Wang M, Zhang X, Zhou Z (2014). On the absence of sternal elements in *Anchiornis* (Paraves) and *Sapeornis* (Aves) and the complex early evolution of the avian sternum. Proceedings of the National Academy of Sciences of the United States of America.

[ref-262] Zhou Z, Barrett PM, Hilton J (2003). An exceptionally preserved Lower Cretaceous ecosystem. Nature.

[ref-263] Zylberberg L, Laurin M (2011). Analysis of fossil bone organic matrix by transmission electron microscopy. Comptes Rendus Palevol.

